# Multifaceted roles of BBX transcription factors: impacts on key agronomical traits and environmental resilience

**DOI:** 10.1111/nph.70997

**Published:** 2026-02-18

**Authors:** Bruno Silvestre Lira, Juliene Moreira, Luciano Freschi, Magdalena Rossi

**Affiliations:** ^1^ Departamento de Botânica, Instituto de Biociências Universidade de São Paulo Rua do Matão 277 São Paulo 05508‐090 Brazil

**Keywords:** B‐box proteins, biotic and abiotic stress, flowering, gene family, photomorphogenesis, shade avoidance, specialized metabolism

## Abstract

B‐box (BBX) proteins were initially characterized as transcription factors connecting light signaling to the regulation of flowering time and seedling photomorphogenesis. However, over the last decade, increasing evidence has shown that they integrate light and hormone signaling, modulating multiple physiological processes during plant life. In this review, we provide an overview of the structure of this protein family and the function of its members in *Arabidopsis thaliana* model species. Then, we specifically discuss the role of BBX factors in controlling agronomically important traits in crop species, including flowering time, fruit and seed production, responses to biotic and abiotic stresses, and specialized metabolism. Finally, we address several aspects that require further investigation to dissect the mechanisms behind the multifaceted roles of BBX proteins and their implications for crop improvement. Current knowledge positions this protein family as a strategic target for the integrated modulation of signal transduction pathways, intended to optimize diverse physiological responses and improve the yield and nutritional value of food feedstocks.

**Table:** 

	Contents	
	Summary	762
I	Introduction	762
II	The BBX protein family: insights from *Arabidopsis thaliana*	763
III	The BBX gene family in crop species	765
IV	Reproductive development	765
V	Stress response	771
VI	Specialized metabolism	774
VII	Conclusions and perspectives	778
	Acknowledgements	781
	References	781

## I. Introduction

As sessile organisms, plants rely on dynamic and highly interconnected signaling networks to adapt to continuously changing environmental conditions. Central to these mechanisms, transcription factor (TF)–controlled gene regulatory networks rapidly reprogram gene expression, thereby enabling metabolic and developmental adjustments throughout the plant life cycle. Among the TF families, the B‐box (BBX) zinc finger proteins contain at least one BBX domain, which encompasses *c*. 40 amino acids and is stabilized by cysteine, histidine and aspartic acid residues along with two zinc ions. Moreover, BBX proteins may contain a CONSTANS, CONSTANS‐like and TIMING OF CAB1 (CCT) domain characterized by its highly conserved 42–43 amino acids. The TF activity of the BBX family members relies on both the BBX and CCT domains. Lastly, a nuclear localization signal occurs in the C‐terminus region of the protein (Song *et al*., 2024a).

BBX proteins regulate the expression of their target genes by recognizing specific *cis*‐regulatory motifs within promoter regions, including the CCAAT box (Ben‐Naim *et al*., 2006), CORE2 (TGTGN_2‐3_ATG, Tiwari *et al*., 2010), CCACA (Gnesutta *et al*., 2017), the G‐box (CACGTG; Song *et al*., 2020) and a modified G‐box motif (TACGTG; Xiong *et al*., 2019). Additionally, BBX proteins frequently form heterodimers with other TFs, thereby either enhancing or repressing their regulatory activity (Cao *et al*., 2023). Meanwhile, BBX proteins are often degraded by E3 ubiquitin ligase complex‐mediated post‐translational regulation (Lin *et al*., 2018). With these modes of action, BBX TFs regulate developmental processes ranging from germination and seedling establishment to flowering and senescence (Song *et al*., 2024a).

Therefore, with a focus on crop species, this review explores how BBX TFs enable plant adaptation bridging environmental stimuli and developmental processes through multifaceted regulatory mechanisms.

## II. The BBX protein family: Insights from *Arabidopsis thaliana*

**Fig. 1:**
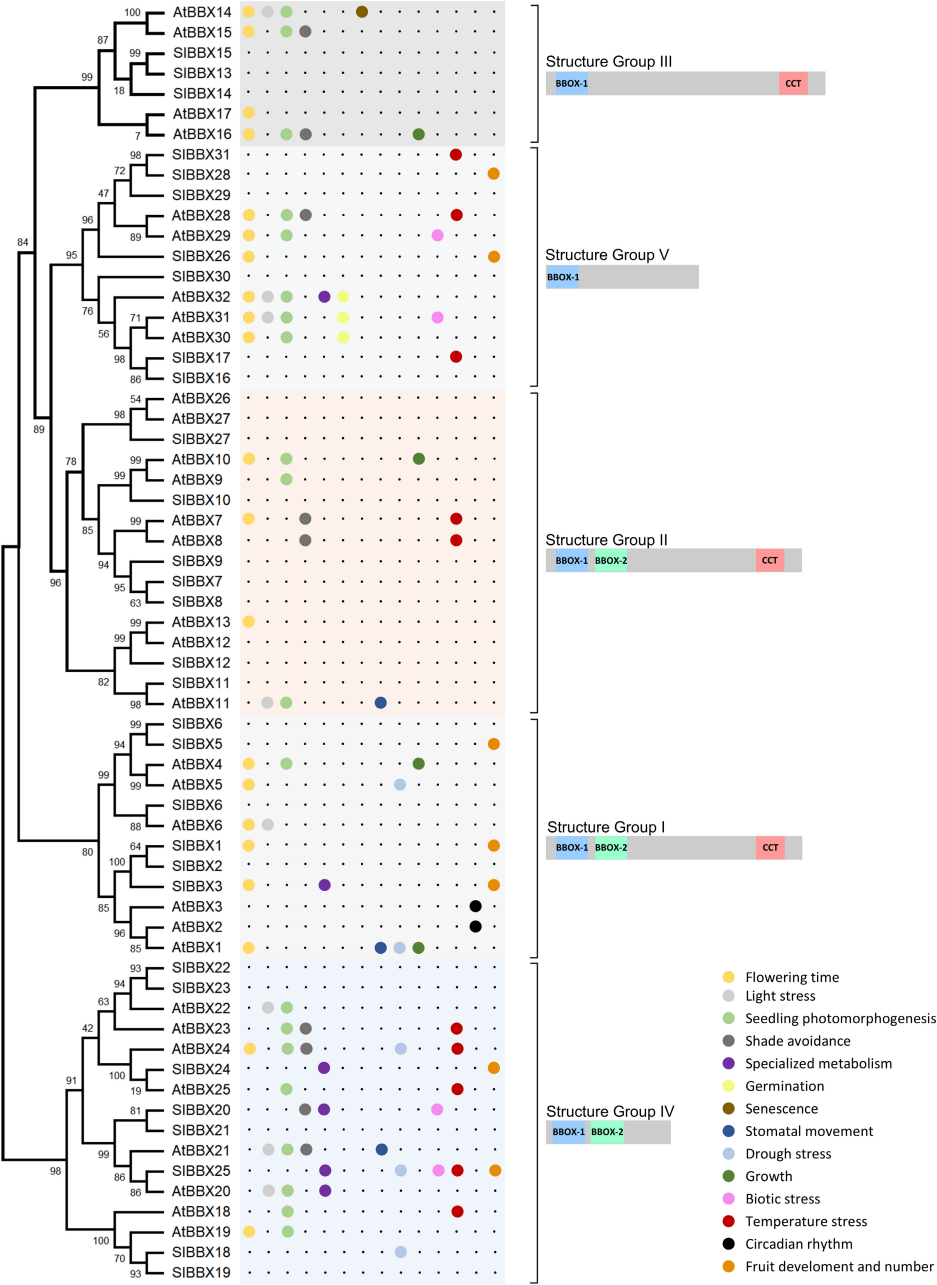
B‐box (BBX) proteins cluster into five structural groups. Phylogenetic analysis of the 32 *Arabidopsis thaliana* and the 31 *Solanum lycopersicum* BBX proteins. Sequences were aligned with the default parameters of the Expresso T‐Coffee algorithm (Armougom *et al*., 2006). The tree was reconstructed from the obtained alignment using the phyml 3.0 package (Guindon *et al*., 2010) with the JTT (Jones‐Taylor‐Thornton) substitution model, and the proportion of invariable sites and gamma shape parameter were estimated from the data sample. Then, the tree was optimized by tree topology and branch length, and improved by subtree pruning and regrafting. Finally, the branch support was calculated by the approximate likelihood‐ratio test Shimodaira–Hasegawa‐like (aLTR SH‐like). The protein topology for each structural group was predicted from the group consensus sequence using the InterPro database (Blum *et al*., 2025) and is shown on the right side. The functions are indicated by colored circles and are listed and referred to in Supporting Information Table S1 for *A. thaliana* and Table 1 for *S. lycopersicum*. The loci were numbered according to Lira *et al*. (2020).

In *A. thaliana*, the 32 AtBBX proteins cluster into five structural groups based on the number and evolutionary origin of BBX domains and on the presence or absence of the CCT domain. Group I and II proteins display two BBX domains with independent origins, and one CCT domain; Group III structure consists of a unique BBX and a CCT domain; lastly, Groups IV and V lack CCT and contain two and one BBX domains, respectively (Khanna *et al*., 2009). AtBBX26 and AtBBX27, initially structural Group V members, were recently reassigned to Group II following a robust phylogenetic analysis and the identification of a second BBX domain and a vestigial CCT domain (Lira *et al*., 2020; Fig. 1).

Out of the 32 *AtBBX* genes, only *AtBBX12*, *AtBBX26* and *AtBBX27* remain functionally uncharacterized (Supporting Information Table S1). Interestingly, 66 and 79% of the 29 characterized AtBBXs are associated with flowering time and light responses (i.e. seedling photomorphogenesis, shade avoidance and light stress), respectively. Additionally, most AtBBXs have been associated with other functions, highlighting the pleiotropic effects of BBX family members on plant physiology. Given the current state of knowledge, no structural groups can yet be reliably correlated with specific physiological processes (Fig. 1).

The first AtBBX characterized was AtBBX1 (CONSTANS, AtCO), which directly binds to the florigen *FLOWERING LOCUS T* (*AtFT*) promoter, inducing vegetative‐to‐reproductive meristem transition (Putterill *et al*., 1995; Tiwari *et al*., 2010). Similarly, AtBBX6/24 also induce *AtFT* expression (Hassidim *et al*., 2009; Li *et al*., 2014), while AtBBX4/5/32 inhibit the florigen transcription (Cheng & Wang, 2005; Tripathi *et al*., 2017; Steinbach, 2019). The other flowering‐associated AtBBXs regulate *AtBBX1*, either repressing its expression (*i.e*. AtBBX7; Cheng & Wang, 2005) or modulating its transcriptional regulatory activity over *AtFT* through physical interaction. The latter is the case of the negative AtBBX10/13/14/15/16/17/19/30/31 (Wang *et al*., 2014; Graeff *et al*., 2016; Ordoñez‐Herrera *et al*., 2018; Xu *et al*., 2022b; Susila *et al*., 2023; Rahul *et al*., 2024) and the positive AtBBX28/29 factors (Wang *et al*., 2021).

Photomorphogenesis is the modulation of plant development in response to variations in light conditions. One extensively studied aspect is seedling photomorphogenesis, the transition from a heterotrophic to a photoautotrophic organism. If light is limiting, the seedling will exhibit elongated hypocotyls with underdeveloped cotyledons; in contrast, upon light exposure, the seedlings will show thick and short hypocotyls with open cotyledons (Nemhauser & Chory, 2002). Twenty AtBBXs have been characterized as seedling photomorphogenesis regulators, participating in an intricate transcriptional and post‐translational regulatory network together with ELONGATED HYPOCOTYL 5 (AtHY5) and/or PHYTOCHROME INTERACTING FACTORS (AtPIFs), which are the light‐signaling master positive and negative TFs, respectively. Among the positive regulators of seedling photomorphogenesis, AtBBX11/14/15/16 induce *AtHY5* expression (Zhao *et al*., 2020; Nasim *et al*., 2024), AtBBX22/23 regulate AtHY5 protein activity through dimerization (X. Zhang *et al*., 2017; Podolec *et al*., 2022), while AtBBX20/21 act at both transcriptional and post‐translational levels (Wei *et al*., 2016; Xu *et al*., 2016; Podolec *et al*., 2022). By contrast, AtBBX4 interacts with AtPIF3, alleviating the AtPIF3‐mediated repression of photomorphogenic genes (Heng *et al*., 2019). Moreover, AtBBX9/14/15/16/20/21 induce photomorphogenesis by downregulating auxins, brassinosteroids (BRs), and gibberellins (GAs) metabolism and signaling (Fan *et al*., 2012; Xu *et al*., 2017; Nasim *et al*., 2024; Song *et al*., 2024b). On the contrary, the negative regulators AtBBX24/25/28/29 physically interact with AtHY5, hindering its ability to bind to its target genes' promoters, thereby inhibiting photomorphogenesis (Gangappa *et al*., 2013; Song *et al*., 2020). Interestingly, AtHY5 represses the expression of *AtBBX30/31*, whose protein products induce *AtBBX28/29*, resulting in a feedback loop that fine‐tunes seedling development (Song *et al*., 2020). The negative effect of AtBBXs on photomorphogenesis is also mediated by hormones. For instance, AtBBX18, which induces hypocotyl elongation by upregulating GA biosynthesis (Wang *et al*., 2011), and AtBBX28/29/32, which regulate BRs signaling (Cao *et al*., 2022; Ravindran *et al*., 2021). Finally, AtBBX19 promotes hypocotyl growth by facilitating AtCOP1‐mediated degradation of EARLY FLOWERING 3 (AtELF3; Wang *et al*., 2014).

AtBBXs also regulate cotyledon development during seedling photomorphogenesis. The GENOMES UNCOUPLED 1 (AtGUN1)/GOLDEN2 LIKE 1 (AtGLK1) retrograde signaling module regulates the expression of *AtBBX14/16*, which are positive regulators of cotyledon development (Veciana *et al*., 2022; Atanasov *et al*., 2024). Moreover, as Chl biosynthetic genes are upregulated by AtBBX11, its expression is tightly regulated by AtPIF3 and AtHY5 to avoid photobleaching (Job & Datta, 2021). AtPIF1‐AtBBX10 dimer inhibits photo‐oxidative damage in de‐etiolating seedlings by repressing Chl biosynthesis in an AtGUN5‐mediated manner in darkness and by inducing antioxidant enzymes under light conditions (L. Zhou *et al*., 2025). Similarly, AtBBX32 acts with AtPIF3, maintaining the repression on AtGUN4/5 and photosynthesis‐associated genes during soil emergence, fine‐tuning cotyledon greening (K. Wang *et al*., 2025).

Shade avoidance syndrome (SAS) is a photomorphogenic response triggered by the reduction in the red : far‐red (R : FR) ratio of incident light. SAS is characterized by hypocotyl, internodes and petiole elongation, hyponasty, reduced branching and early flowering (Ballaré & Pierik, 2017). The overexpression of *AtBBX16* results in longer hypocotyls and reduced branching under shade conditions (Wang *et al*., 2013). AtBBX24 also positively regulates SAS, which, through the interaction with the GA repressors AtDELLAs, releases AtPIF4 to induce the expression of auxin signaling genes (Crocco *et al*., 2015). AtBBX28 triggers SAS by inducing auxin‐related genes in an AtCOP1‐dependent manner (Saura‐Sánchez *et al*., 2024). Finally, AtBBX7/8 positively regulate the low R : FR‐induced *AtPIF4* expression and promote hypocotyl growth (Bian *et al*., 2025). Conversely, AtBBX21 reduces BRs biosynthesis, repressing hypocotyl elongation under shade conditions (Gómez‐Ocampo *et al*., 2024).

The role of several AtBBX proteins in plant stress responses has been increasingly attracting attention. AtBBX1 antagonizes ABSCISIC ACID‐RESPONSIVE ELEMENT BINDING FACTORS (AtABFs) through dimerization, downregulating salinity‐responsive genes and, consequently, stress tolerance (Du *et al*., 2023). AtBBX21/24 negatively regulate *ABSCISIC ACID INSENSITIVE 5* (*AtABI5*) expression, reducing abscisic acid (ABA) sensitivity during germination (Xu *et al*., 2014; Chiriotto *et al*., 2023). Similarly, *Atbbx5* exhibits increased sensitivity to ABA and salt stress during seed germination (Min *et al*., 2015).

AtBBX11/31 induce photoprotection and DNA damage repair genes under UV‐B light stress (Yadav *et al*., 2019; Job *et al*., 2022). Similarly, AtBBX20/21/22 are needed for hypocotyl growth inhibition and photoprotective pigment accumulation in response to UV‐B (Podolec *et al*., 2022). AtBBX14 is required for acclimation to light‐stress conditions, as *Atbbx14* plants display compromised photosynthetic activity when exposed to high light (Atanasov *et al*., 2024). By contrast, AtBBX32 impairs light‐stress acclimation by downregulating photosynthesis‐associated gene expression under high‐light conditions (Alvarez‐Fernandez *et al*., 2021).

Finally, the AtPIF4‐mediated hypocotyl elongation triggered by high temperature is induced by the partially redundant AtBBX18/23/24/25 (Ding *et al*., 2018; Malakar *et al*., 2025b) and repressed by AtBBX21 (Malakar *et al*., 2025a). A recent report advanced the limits of current understanding by highlighting the importance of both sense and antisense *AtBBX28* transcript expression in enhancing plant freezing tolerance (Meena *et al*., 2024). Finally, under cold stress, blue light‐induced phosphorylation stabilizes CRYPTOCHROME 2 (AtCRY2), which interacts with AtCOP1, preventing AtHY5 degradation. As direct targets of AtHY5, AtBBX7/8 positively regulate freezing tolerance by modulating cold‐responsive genes (Li *et al*., 2021).

## III. The BBX gene family in crop species

A gene family is a group of genes that arose from duplication of a single ancestral gene and have retained sequence similarities. In plants, gene families expand through whole‐genome (WGD), single‐gene and segmental duplication events, which enhance genetic diversity and provide raw material for emergence of new functions (Fang *et al*., 2022). Within plant genomes, tandem gene duplication is a major single‐gene duplication mechanism, characterized by the occurrence of two or more adjacent homologous genes. Yet, segmental duplications arise from the duplication of larger genomic fragments containing variable numbers of genes that are identified as syntenic blocks. By surveying 18 representative plant species, Yu *et al*. (2022) proposed that the expansion of the *BBX* gene family is primarily driven by WGD, with segmental duplication acting as a secondary contributing mechanism.

Whole‐genome surveys over the last decade have identified BBX gene families in 79 plant species, most of which contain between 16 and 40 members. In all cases, the loci cluster into the five structural groups originally described (Table S2). In some species, the copy number is notably higher. Such is the case of cotton (*Gossypium hirsutum*), which has 127 BBX proteins, mainly originated from lineage‐specific WGD events (Paterson *et al*., 2012; Sun *et al*., 2023). Moreover, the number of BBX‐coding genes in the genomes also reflects the ploidy of the species. In strawberry, while the diploid species (*Fragaria vesca*) contains 22 *BBX* genes, 51 are found in the polyploid genotype (*F. ananassa*; D. Xu *et al*., 2023; Ye *et al*., 2021). In this line, the tetraploid genome of *Vaccinium corymbosum* blueberry harbors 83 *BBX* genes, while the diploid *V. darrowii* has only 24 loci (Xue *et al*., 2025). The number of *BBX* genes in the genomes of the polyploid species *Brassica napus*, *B. carinata*, *B. juncea* and wheat (*Triticum aestivum*) is approximately three times that of the number of loci in *A. thaliana* (Chen *et al*., 2021; Singh *et al*., 2021; Zheng *et al*., 2021).

Most genome‐wide BBX identification studies also characterize their expression patterns showing that these genes are differentially expressed across organs and in response to hormonal and stress treatments. Based on this information, subsequent studies have investigated the mechanisms through which BBX proteins modulate agronomically important traits in crop species, including reproductive development, abiotic and biotic stress responses, and specialized metabolism. Below, we review the current knowledge on the involvement of BBX proteins in these processes.

## IV. Reproductive development

### 1. Flowering time

**Fig. 2:**
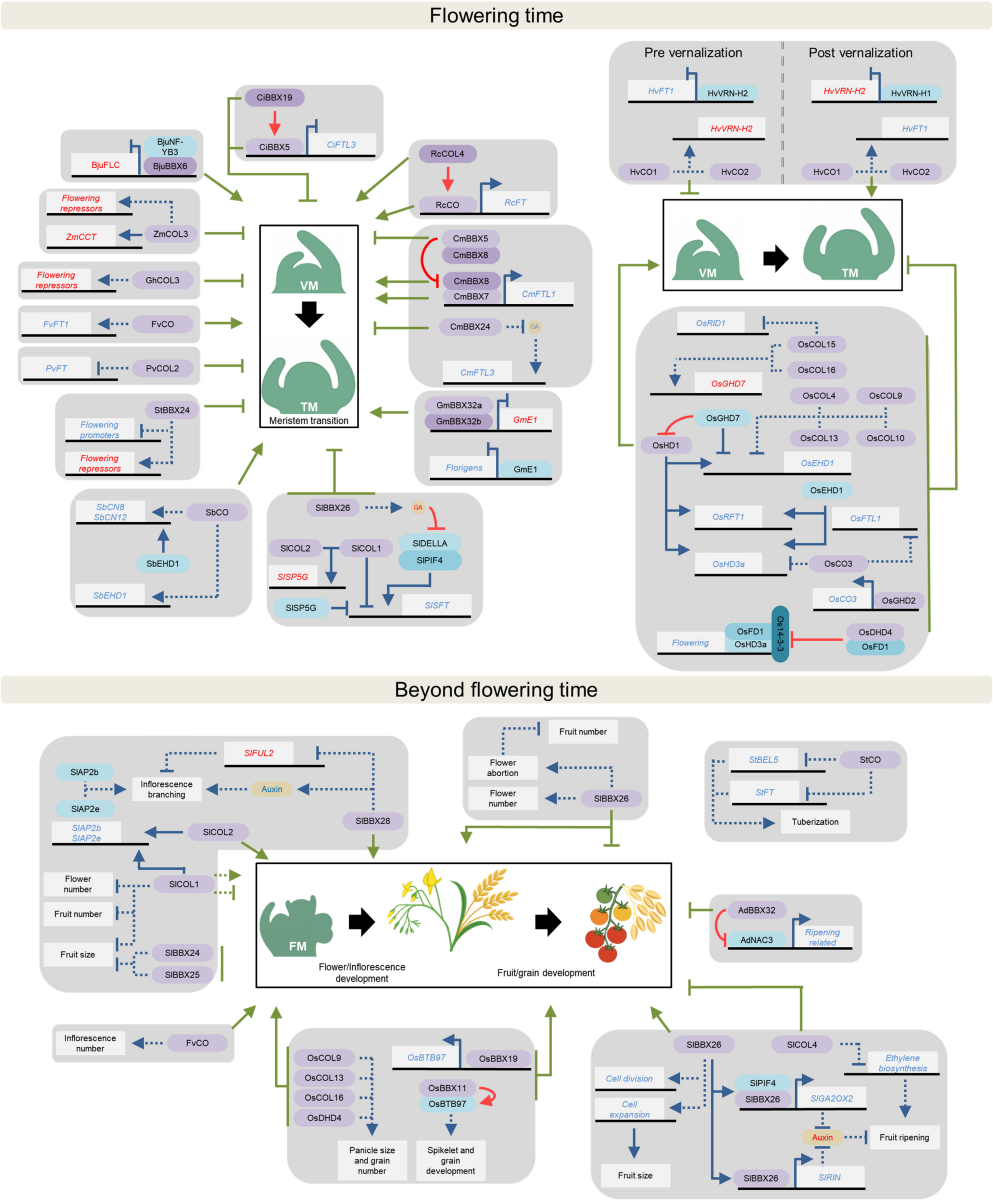
B‐box (BBX) genes that regulate reproductive development in crop species. The species are indicated by the genus and species initials: *Actinidia deliciosa* (*Ad*), *Brassica juncea* (*Bju*), *Chrysanthemum indicum* (*Ci*), *Chrysanthemum morifolium* (*Cm*), *Fragaria vesca* (*Fv*), *Glycine max* (*Gm*), *Gossypium hirsutum* (*Gh*), *Hordeum vulgaris* (*Hv*), *Oryza sativa* (*Os*), *Phaseolus vulgaris* (*Pv*), *Rosa chinensis* (*Rc*), *Solanum lycopersicum* (*Sl*), *Solanum tuberosum* (*St*), *Sorghum bicolor* (*Sb*) and *Zea mays* (*Zm*). Black lines and white boxes represent DNA and genes, respectively. BBX proteins are represented in purple, other proteins in blue and hormones in yellow. Blue lines indicate transcriptional regulation, red lines indicate post‐translational regulation, and green lines indicate the effect of the BBX protein on the phenotype. Arrows represent positive regulation, and blunt‐ended arrows represent inhibition. Solid and dotted lines indicate direct or indirect effects, respectively. Both gene and hormone names are written in red or blue, depending on whether they have a negative or a positive effect on the phenotype, respectively. Gene, protein and hormone abbreviations are detailed in the text. VM, TM and FM: vegetative, transition and floral meristem, respectively.

The first function identified for BBX proteins was the regulation of flowering time. As a key determinant of crop yield in many species, this discovery prompted investigations into whether crop BBXs regulate flowering through analogous mechanisms (Fig. 2; Table 1). In this sense, by recovering *Atco* late flowering phenotype, BBXs were identified as positive regulators of flowering, including canola (*Brassica napus*) BnCOa1 (Robert *et al*., 1998), cotton (*G. hirsutum*) GhCOL1A and GhCOL1D (Cai *et al*., 2017), peach (*Prunus persica*) PpCO (Zhang *et al*., 2015), ryegrass (*Lolium perenne*) LpCO (Martin *et al*., 2004), and soybean (*Glycine max*) GmCOL5 (Fan *et al*., 2014).

**Table 1:** Functionally characterized *BBX loci* in crop species.

Species	Common name	BBX^1^	Function	References
**Reproductive development**				
*Flowering time*				
*Brassica juncea*	Chinese mustard	BjuBBX6	Positive	Feng *et al*. (2025)
*Brassica napus*	Canola	BnCOa1	Positive	Robert *et al*. (1998)
*Chrysanthemum indicum*	Chrysanthemum	CiBBX5	Negative	C. Yang *et al*. (2025)
		CiBBX19	Negative	C. Yang *et al*. (2025)
*Chrysanthemum morifolium*	Chrysanthemum	CmBBX5	Negative	Wang *et al*. (2024)
		CmBBX7	Positive	Zhai *et al*. (2023)
		CmBBX8	Positive	Wang *et al*. (2020)
				Zhai *et al*. (2023)
		CmBBX24	Negative	Y. Yang *et al*. (2014)
*Fragaria vesca*	Strawberry	FvCO	Positive	Kurokura *et al*. (2017)
*Glycine max*	Soybean	GmCOL5	Positive	Fan *et al*. (2014)
		GmBBX32a	Negative	C. Gao *et al*. (2025)
		GmBBX32b	Negative	C. Gao *et al*. (2025)
*Gossypium hirsutum*	Cotton	GhCOL1A	Positive	Cai *et al*. (2017)
		GhCOL1D	Positive	Cai *et al*. (2017)
		GhCOL3	Negative	Z. Song *et al.* (2025)
*Hordeum vulgare*	Barley	HvCO1	Positive	Mulki & von Korff (2016)
		HvCO2	Positive	Mulki & von Korff (2016)
*Lolium perenne*	Ryegrass	LpCO	Positive	Martin *et al*. (2004)
*Oryza sativa*	Rice	OsCO3	Negative	Kim *et al*. (2008)
		OsCOL4	Negative	Lee *et al*. (2010)
		OsCOL9	Negative	H. Liu *et al*. (2016)
		OsCOL10	Negative	Tan *et al*. (2016)
		OsCOL13	Negative	Sheng *et al*. (2016)
		OsCOL15	Negative	Wu *et al*. (2018)
		OsCOL16	Negative	Wu *et al*. (2017)
		OsDHD4	Negative	Cai *et al*. (2021)
		OsGhd2	Negative	Fan *et al*. (2023)
		OsHd1	Positive	Z. Zhang *et al*. (2017)
*Phaseolus vulgaris*	common bean	PvCOL2	Negative	González *et al*. (2021)
*Prunus persica*	Peach	PpCO	Positive	Zhang *et al*. (2015)
*Rosa chinensis*	Rose	RcCO	Positive	Lu *et al*. (2020)
		RcCOL4	Positive	Lu *et al*. (2020)
*Solanum lycopersicum*	Tomato	SlBBX1(SlCOL2)	Negative	J. Song *et al*. (2025)
		SlBBX3(SlCOL1)	Negative	Cui *et al*. (2022)
				J. Song *et al*. (2025)
		SlBBX26	Negative	Moreira *et al*. (2025)
*Solanum tuberosum*	Potato	StBBX24	Negative	Kiełbowicz‐Matuk *et al*. (2022)
*Sorghum bicolor*	Sorghum	SbCO	Positive	S. Yang *et al*. (2014)
*Zea mays*	Maize	ZmCOL3	Negative	Jin *et al*. (2018)
*Beyond flowering time*				
*Actinidia deliciosa*	Kiwi	AdBBX32	Negative (fruit ripening)	Y. Yang *et al*. (2025)
*Fragaria vesca*	Strawberry	FvCO	Positive (inflorescence number)	Kurokura *et al*. (2017)
*Oryza sativa*	Rice	OsBBX11	Positive (harvestable organs development)	Shalmani *et al*. (2023)
		OsBBX19	Positive (harvestable organs development)	Shalmani *et al*. (2023)
		OsCOL9	Positive (harvestable organs number)	H. Liu *et al*. (2016)
		OsCOL13	Positive (harvestable organs number)	Sheng *et al*. (2016)
		OsCOL16	Positive (harvestable organs number)	Wu *et al*. (2017)
		OsDHD4	Positive (harvestable organs number)	Cai *et al*. (2021)
*Solanum lycopersicum*	Tomato	SlBBX1(SlCOL2)	Positive (harvestable organs number)	J. Song *et al*. (2025)
		SlBBX3(SlCOL1)	Negative (harvestable organs number and size)	Cui *et al*. (2022)
			Positive (inflorescence branching)	J. Song *et al*. (2025)
		SlBBX5(SlCOL4)	Negative (fruit ripening)	Wu *et al*. (2025)
		SlBBX24	Negative (harvestable organs number and size)	Cui *et al*. (2022)
		SlBBX25	Negative (harvestable organs size)	Luo et al. (2023)
		SlBBX26	Positive (fruit ripening)	Moreira *et al*. (2025)
			Negative (harvestable organs size)	Moreira *et al*. (2025)
		SlBBX28	Positive (harvestable organs number)	Lira *et al*. (2022)
*Solanum tuberosum*	Potato	StCO	Negative (harvestable organs number)	González‐Schain *et al*. (2012)
**Stress response**				
*Abiotic stress*				
Cold tolerance				
*Malus domestica*	Apple	MdBBX37	Positive	An *et al*. (2021a)
*Nicotiana tabacum*	Tabacco	NtBBX9	Negative	S. Liu *et al*. (2023)
		NtBBX11	Positive	S. Liu *et al*. (2023)
*Solanum lycopersicum*	Tomato	SlBBX17	Positive	Song *et al*. (2023)
		SlBBX25	Positive	Ma *et al*. (2025)
		SlBBX31	Positive	Zhu *et al*. (2023)
*Solanum tuberosum*	Potato	StBBX14	Positive	Zhang *et al*. (2025a)
Drought tolerance				
*Arachis hypogaea*	Peanut	AhBBX6	Positive	Tang *et al*. (2024)
*Brassica juncea*	Chinese mustard	BjuBBX6	Negative	Feng *et al*. (2025)
*Brassica napus*	Canola	BnBBX22.A07	Positive	Zhang *et al*. (2024)
*Chrysanthemum morifolium*	Chrysanthemum	CmBBX19	Negative	Xu *et al*. (2020)
*Glycine max*	Soybean	GmCOL1a	Positive	C. Xu *et al*. (2023)
*Malus domestica*	Apple	MdBBX7(MdCOL9)	Positive	Chen *et al*. (2022)
		MdBBX22	Positive	An *et al*. (2021b)
*Oryza sativa*	Rice	OsGhd2	Negative	J. Liu *et al*. (2016)
		OsBBX11	Positive	Lei *et al*. (2023)
*Solanum lycopersicum*	Tomato	SlBBX18	Positive	Li *et al*. (2024)
		SlBBX25	Positive	Ma *et al*. (2024)
*Solanum tuberosum*	Potato	StBBX24	Positive	Kiełbowicz‐Matuk *et al*. (2022)
Heat tolerance				
*Solanum lycopersicum*	Tomato	SlBBX17	Positive	Xu *et al*. (2022a)
Shade avoidance response				
*Brassica juncea*	Chinese mustard	BjuCOL13	Positive	Muntha *et al*. (2019)
*Solanum lycopersicum*	Tomato	SlBBX20	Negative	Shiose *et al*. (2024)
*Zea mays*	Maize	ZmDBB2	Positive	X. Wang *et al*. (2025)
*Biotic stress*				
*Bambusa pervariabilis × Dendrocalamopsis grandis*	Bamboo	BDBBX21	Positive (fungi resistance)	Y. Liu *et al*. (2024)
*Capsicum annuum*	Pepper	CaBBX14	Positive (fungi resistance)	Zhou *et al*. (2023)
*Chrysanthemum morifolium*	Chrysanthemum	CmBBX32	Positive (fungi resistance)	B. Wang *et al*. (2025)
*Cucumis sativus*	Cucumber	CsCOL9	Positive (white fly resistance)	Xie *et al.* (2025)
*Ipomoea batatas*	Sweet potato	IbBBX24	Positive (fungi resistance)	Zhang *et al*. (2020)
*Lilium pumilum*	Lily	LpBBX28	Positive (trichome initiation)	Xin *et al*. (2025)
*Solanum lycopersicum*	Tomato	SlBBX20	Positive (fungi resistance)	Shiose *et al*. (2024)
		SlBBX25	Negative (fungi resistance)	Luo *et al*. (2023)
*Solanum tuberosum*	Potato	StBBX27	Positive (oomycete resistance)	Sun *et al*. (2025)
**Specialized metabolism**				
*Artemisia annua*	Sweet wormwood	AaBBX21	Positive (artemisinin in vegetative organs)	He *et al*. (2024)
*Cannabis sativa*	Cannabis	CsaCOL1	Positive (cannabinoids in hairy roots)	M. Gao *et al*. (2025)
		CsaCOL5	Positive (cannabinoids in hairy roots)	M. Gao *et al*. (2025)
		CsaCOL7	Positive (cannabinoids in hairy roots)	M. Gao *et al*. (2025)
*Capsicum annuum*	Pepper	CaBBX10	Positive (chlorophyll and carotenoids in fruits)	J. Wang *et al*. (2025)
		CaBBX20	Positive (carotenoids in fruits)	Ma *et al*. (2023)
*Chrysanthemum morifolium*	Chrysanthemum	CmBBX28	Negative (anthocyanins in flowers )	L.J. Zhou *et al*. (2025)
		CmBBX20	Positive (flavonoids and chlorogenic acid in flowers)	Lu *et al*. (2024)
*Citrus* spp.	Orange, pomelo	ChBBX22	Positive (carotenoids and anthocyanins in fruits)	Fu *et al*. (2024)
*Citrus sinensis*	Blood orange	CsBBX24	Positive (carotenoids and anthocyanins in fruits)	Fu *et al*. (2025)
*Fragaria x ananassa*	Strawberry	FaBBX22	Positive (anthocyanins in fruits)	Liu *et al*. (2022)
		FaBBX24	Positive (anthocyanins in fruits)	Zhang *et al*. (2023)
*Glycine max*	Soybean	GmBBX4	Negative (anthocyanins in seeds )	Song *et al*. (2024c)
		GmBBX22	Positive (flavonoids/anthocyanins in vegetative organs)	Zhan *et al*. (2025)
*Gossypium hirsutum*	Cotton	GhBBX21	Positive (anthocyanins in vegetative organs)	Li *et al*. (2025b)
		GhBBX24(GhDR)	Negative (anthocyanins in vegetative organs)	X. Wang *et al*. (2023)
				Li *et al*. (2025b)
*Ipomoea batatas*	Sweeto potato	IbBBX29	Positive (flavonoids in leaves and roots)	Gao *et al*. (2023)
*Lilium* spp.	Lily	LvBBX24	Positive (anthocyanins in flowers )	Gao *et al*. (2024)
*Malus domestica*	Apple	MdCOL4	Negative (anthocyanins in fruits )	Fang *et al*. (2019a)
		MdCOL6	Positive (anthocyanins in fruits )	Wang *et al*. (2023b)
		MdBBX20	Positive (anthocyanins in fruits )	Fang *et al*. (2019b)
		MdBBX21	Positive (anthocyanins in fruits )	Zhang *et al*. (2021)
		MdBBX22	Positive (anthocyanins in fruits )	An *et al*. (2019)
				Zhang *et al*. (2022)
		MdBBX37	Negative (anthocyanins in leaves)	An *et al*. (2020)
*Mangifera indica*	Mango	MiBBX24	Positive (anthocyanins and carotenoids in fruits)	Pan *et al*. (2025)
		MiBBX27	Positive (anthocyanins and carotenoids in fruits)	Pan *et al*. (2025)
*Petunia hybrida*	Petunia	PhCOL16a	Positive (chlorophyll in flowers)	Ohmiya *et al*. (2019)
*Prunus avium*	Sweet cherry	PavBBX6	Positive (anthocyanins in fruits )	Wang *et al*. (2023a)
		PavBBX9	Positive (anthocyanins in fruits )	Wang *et al*. (2023a)
*Prunus persica*	Peach	PpBBX32	Positive (anthocyanins in fruits )	Huang *et al*. (2024)
*Pyrus pyrifolia x Pyrus communis*	Pear	PyBBX16	Positive (anthocyanins in fruits )	Bai *et al*. (2019a)
		PyBBX18	Positive (anthocyanins in fruits )	Bai *et al*. (2019b)
		PyBBX21	Negative (anthocyanins in fruits )	Bai *et al*. (2019b)
		PyBBX24	Negative (anthocyanins in fruits and vegetative organs)	Li *et al*. (2025a)
				Yang *et al*. (2024)
*Rubus chingii*	Palmleaf raspberry	RcBBX26	Positive (anthocyanins in leaves)	Xu *et al*. (2024)
*Solanum lycopersicum*	Tomato	SlBBX3(SlCOL1)	Positive (anthocyanins in vegetative organs)	Liu *et al*. (2025)
			Positive (chlorophylls in fruits)	Cui *et al*. (2025)
		SlBBX20	Positive (flavonoids in fruits)	Shiose *et al*. (2024)
		SlBBX24	Positive (anthocyanins in fruits)	He *et al*. (2023)
			Positive (chlorophylls in fruits)	Cui *et al*. (2025)
		SlBBX25	Positive (carotenoids and flavonoids in fruits)	Xiong *et al.* (2019)
			Positive (anthocyanins in vegetative organs)	Luo *et al*. (2021)
*Solanum melongena*	Eggplant	SmBBX22	Positive (anthocyanins in fruits)	J. Li *et al*. (2025)
*Triticum aestivum*	Wheat	TaBBX3B	Positive (anthocyanins in seeds)	Jiang *et al*. (2023)
*Vitis vinifera*	Grape	VvBBX8	Positive (anthocyanins in fruits)	Qiu *et al*. (2025)
		VvBBX11	Positive (anthocyanins in fruits)	Qiu *et al*. (2025)
		VvBBX32	Positive (anthocyanins in fruits)	Qiu *et al*. (2025)
		VvBBX44	Negative (anthocyanins in fruits)	W. Liu *et al*. (2023)

^1^ All acronyms are detailed in the main text.

In potato (*Solanum tuberosum*), StBBX24 was identified as a repressor of floral transition, as knockdown plants exhibited early flowering and up‐ and downregulation of key flowering positive and negative regulators, respectively (Kiełbowicz‐Matuk *et al*., 2022). Similarly, silencing *GhCOL3* in cotton plants resulted in an early‐flowering phenotype likely acting upstream of florigen repressors (Z. Song *et al*., 2025).

For other BBXs, the molecular mechanisms underlying the regulation of flowering time have been more extensively explored. In the *Brassica juncea* (Chinese mustard), BjuBBX6 interacts with the NUCLEAR FACTOR YB3 (BjuNF‐YB3) and, alone or as a dimer, downregulates the expression of the flowering suppressor *FLOWERING LOCUS C* (*BjuFLC*; Feng *et al*., 2025). The strawberry FvCO, in a long‐day‐flowering accession, controls the expression of *FvFT1*, regulating the differentiation of inflorescence‐producing branch crowns, rather than into vegetative runners (Kurokura *et al*., 2017). In *Sorghum bicolor*, SbCO not only induces the florigens *CENTRORADIALIS 8* (*SbCN8*) and *SbCN12*, but also activates the expression of the florigens activator *EARLY HEADING DATE 1* (*SbEHD1; S*. Yang *et al*., 2014).

The three tomato (*Solanum lycopersicum*) BBXs characterized as flowering regulators act as repressors. SlCOL1 and SlCOL2 inhibit the expression the florigen *SINGLE FLOWER TRUSS* (*SlSFT*), either directly (Cui *et al*., 2022) or inducing *SELF PRUNNING 5G* (*SlSP5G*) flowering repressor (J. Song *et al*., 2025). SlBBX26, on the contrary, indirectly regulates flowering by promoting the accumulation of GAs, which disrupt the *SlSFT* inducer SlPIF4‐SlDELLA dimer (Moreira *et al*., 2025).

In *Chrysanthemum morifolium*, the long‐day‐associated florigen *FLOWERING LOCUS T LIKE 1* (*CmFTL1*) is directly activated by the synergistic action of CmBBX7 and CmBBX8 (Wang *et al*., 2020; Zhai *et al*., 2023). Conversely, CmBBX5 dimerizes with CmBBX8, reducing the transcriptional promoting activity of the latter (Wang *et al*., 2024). The short‐day florigen *CmFTL3* is indirectly repressed by CmBBX24, as this protein inhibits GA synthesis, which is a florigen‐inductor hormone in this species (Y. Yang *et al*., 2014). In another chrysanthemum species, *C. indicum*, CiBBX5 directly represses *CiFTL3* expression, which is further strengthened through heterodimerization with CiBBX19 (C. Yang *et al*., 2025).

In the common bean (*Phaseolus vulgaris*), PvCOL2 represses *PvFT* expression under noninductive long‐day conditions. Mutations in *PvCOL2* reduce photoperiod sensitivity, and when combined with loss‐of‐function alleles of *PHYTOCHROME A3* (*PvPHYA3*), result in photoperiod‐insensitive flowering in domesticated cultivars (González *et al*., 2021). Similarly, continuous flowering in rose (*Rosa chinensis*) relies on the interplay between RcCO and RcCOL4. Under short days, RcCO is upregulated and activates RcFT. Under long days, RcCOL4 is induced and, by dimerizing with RcCO, increases its affinity for the RcFT promoter (Lu *et al*., 2020). In soybean, under long day, the GmBBX32a‐GmBBX32b dimer inhibits the transcription of the flowering negative regulator *GmE1* (C. Gao *et al*., 2025).

In maize (*Zea mays*), ZmCOL3 directly promotes the expression of flowering repressors, such as *ZmCCT*. Interestingly, polymorphisms in the *ZmCOL3* promoter and in the 5′ untranslated region may have contributed to the adaptation from tropical to temperate regions (Jin *et al*., 2018). Similarly, in the Japanese morning glory (*Ipomoea nil*), it has been suggested that a single base deletion in *InCO* was responsible for an early‐flowering QTL, which might have facilitated adaptation to temperate Asian regions (Katsuyama *et al*., 2025).

To date, rice (*Oryza sativa*) is the crop species with the largest number of BBX proteins identified as flowering regulators. The vegetative‐to‐reproductive growth transition is triggered by EARLY HEADING DATE 1 (OsEHD1), which activates the expression of the florigens *HEADING DATE 3A* (*OsHD3a*) and *RICE FLOWERING LOCUS T 1* (*OsRFT1*). Conversely, *OsEHD1* transcript accumulation is downregulated by GRAIN NUMBER, PLANT HEIGHT AND HEADING DATE 7 (OsGHD7; J. Song *et al*., 2024). The only known rice BBX that positively regulates flowering is OsHD1, which upregulates *OsEHD1*, *OsHD3a* and *OsRFT1*. The transcriptional activity of this BBX is, however, suppressed through dimerization with OsGHD7 (Z. Zhang *et al*., 2017). Nine OsBBXs are flowering repressors, although it remains unclear whether some act directly or indirectly. Notably, *OsGHD7* is upregulated by OsCOL15 (Wu *et al*., 2018) and OsCOL16 (Wu *et al*., 2017). Additionally, *OsEHD1* is downregulated by OsCOL4 (Lee *et al*., 2010), OsCOL9 (H. Liu *et al*., 2016), OsCOL10 (Tan *et al*., 2016) and OsCOL13 (Sheng *et al*., 2016). Additionally, the BBX OsGHD2 directly upregulates *OsCO3* (Fan *et al*., 2023), which in turn represses the florigen‐encoding genes *OsHD3a* and *OsFTL1* (Kim *et al*., 2008). Moreover, the BBX protein DELAYED HEADING DATE 4 (OsDHD4) interacts with the TF FLOWERING LOCUS D1 (OsFD1), disrupting the florigen activation complex composed of OsHD3a, Os14‐3‐3 and OsFD1 (Cai *et al*., 2021). Furthermore, OsCOL15 inhibits the expression of the flowering activator *RICE INDETERMINATE 1* (*OsRID1*; Wu *et al*., 2018).

Finally, an interesting regulatory mechanism has been identified in barley (*Hordeum vulgaris*), where HvCO1 and HvCO2 may either repress or promote the flowering transition (Mulki & von Korff, 2016). Before vernalization, both BBXs induce *VERNALIZATION2* (*HvVRN*‐*H2*) expression, which downregulates the florigen *HvFT1*. During vernalization, the accumulation of HvVRN‐H1 represses *HvVRN‐H2*, altering the activity of HvCO1 and HvCO2 that upregulate *HvFT1* expression, promoting flowering.

### 2. Beyond flowering time

While the regulation of BBXs over flowering induction has been extensively studied, less is known about their roles in reproductive processes beyond flowering time (Fig. 2; Table 1). Some of the flowering time regulators mentioned above have been reported to affect other reproductive traits. For instance, FvCO positively controls the number of inflorescences per plant (Kurokura *et al*., 2017).

In rice, delaying flowering time through the overexpression of *OsCOL13* (Sheng *et al*., 2016), *OsCOL16* (Wu *et al*., 2017) and *OsDHD4* (Cai *et al*., 2021) increased panicle size, grain number and yield. Conversely, these parameters were reduced in *Oscol9* mutant, even though no differences were detected in overexpressing genotypes (H. Liu *et al*., 2016). For most of these genes, the increased yield was interpreted as a consequence of the prolonged vegetative phase, without further investigation into whether these BBXs also affect panicle development. Furthermore, Shalmani *et al*. (2023) identified OsBBX19 and OsBBX11 as positive regulators of spikelet size and seed production through the transcriptional and post‐translational positive regulation of BROAD‐COMPLEX, TRAM TRACK and BRIC‐A‐BRAC (OsBTB97), respectively.

To date, tomato is the crop species with the highest number of BBXs identified as regulators of the reproductive development beyond flowering transition. SlBBX28 regulates inflorescence branching and fruit number per plant by upregulating auxin biosynthesis and by repressing the expression of the inflorescence branching inhibitor *FRUITFULL 2* (*SlFUL2*; Lira *et al*., 2022). Conflicting results have been reported regarding the effect of SlCOL1 in flowers and fruit production. While Cui *et al*. (2022) describe this gene as a negative regulator of fruit size and flowers and fruits number, J. Song *et al*. (2025) showed that SlCOL1 and SlCOL2 promote inflorescence branching. Interestingly, the overexpression of *SlBBX24*, a SlCOL1‐interacting protein, reduces fruit size (Cui *et al*., 2022). Furthermore, SlBBX25 (as originally Solyc01g110180 locus was named by Chu *et al*., 2016) also negatively regulates fruit size and number (Luo *et al*., 2023). SlBBX26 is another regulator of processes beyond flowering time. Although SlBBX26 deficiency reduced the abortion rate, increasing fruit production, fruits were smaller due to the downregulation of cell division‐ and expansion‐related genes. SlBBX26 also promotes fruit ripening by lowering auxin levels through two coordinated mechanisms. During preripening stages, the SlBBX26–SlPIF4 complex directly upregulates *GA2 OXIDASE 2* (*SlGA2OX2*), promoting GA degradation that reduces auxin accumulation. In addition, SlBBX26 directly activates the master ripening TF *RIPENING INHIBITOR* (*SlRIN*), which diminishes auxin concentrations (Moreira *et al*., 2025). On the contrary, SlCOL4 inhibits fruit ripening by downregulating ethylene biosynthesis‐related genes (Wu *et al*., 2025).

In kiwi (*Actinidia deliciosa*), AdBBX32 interacts with the ripening‐promoting TF NAM, ATAF1/2 and CUC2 3 (AdNAC3), impairing its activity and, consequently, inhibiting ripening‐related genes (Y. Yang *et al*., 2025). Lastly, potato StCO reduces tuberization in repressive and weakly inductive photoperiods by downregulating the tuberization‐inducers *BEL1‐LIKE PROTEIN 5* (*StBEL5*) and *StFT* expression (González‐Schain *et al*., 2012).

## V. Stress response

### 1. Abiotic stress response

**Fig. 3:**
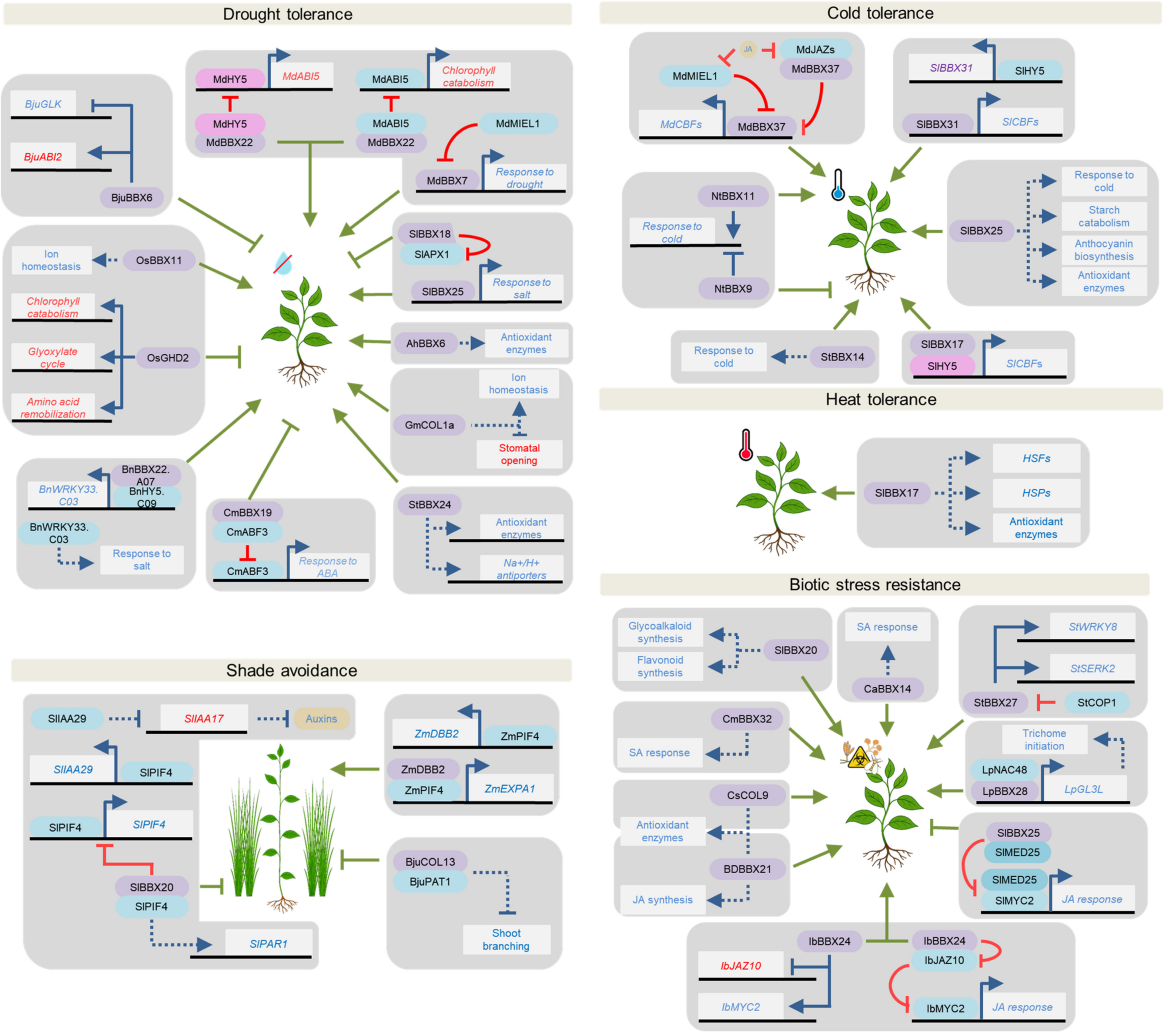
B‐box (BBX) proteins involved in stress‐response regulation in crop species. The species are indicated by the genus and species initials. *Arachis hypogaea* (*Ah*), *Bambusa pervariabilis × Dendrocalamopsis grandis* (*BD*), *Brassica juncea* (*Bju*), *Brassica napus* (*Bn*), *Capsicum annuum* (*Ca*), *Chrysanthemum morifolium* (*Cm*), *Cucumis sativus* (*Cs*), *Glycine max* (*Gm*), *Ipomoea batatas* (*Ib*), *Lilium pumilum* (*Lp*), *Malus domestica* (*Md*), *Nicotiana tabacum* (*Nt*), *Oryza sativa* (*Os*), *Solanum lycopersicum* (*Sl*), *Solanum tuberosum* (*St*) and *Zea mays* (*Zm*). Black lines and white boxes represent DNA and genes, respectively. BBX proteins are represented in purple, HY5 in pink, other proteins in blue and hormones in yellow. Blue lines indicate transcriptional regulation, red lines indicate post‐translational regulation and green lines indicate the effect of the BBX protein on the phenotype. Arrows represent positive regulation and blunt‐ended arrows represent inhibition. Solid and dotted lines indicate direct or indirect effects, respectively. Both gene and hormone names are written in red or blue depending on whether they have a negative or a positive effect on the phenotype, respectively. Gene, protein and hormone abbreviations are detailed in the text.

Throughout the life cycle, plants are exposed to diverse environmental stressors, including drought, salinity, extreme temperatures and fluctuations in light conditions. These abiotic challenges can strongly impair development and yield, especially as climate change increases their frequency and severity (Zhang *et al*., 2025b). To cope with these conditions, plants have evolved complex adaptive strategies, in which BBX proteins have emerged as key regulators (Bandara *et al*., 2022; Fig. 3; Table 1).

In rice, the BBX OsGHD2 negatively regulates drought tolerance by promoting leaf senescence. It directly controls the expression of the Chl catabolism‐related genes *STAY GREEN 1* (*OsSGR1*), *NON‐YELLOW COLORING 1* (*OsNYC3*) and *PHEIDE A OXYGENASE* (*OsPAO*); the glyoxylate cycle enzyme encoding genes *ISOCITRATE LYASE* (*OsICL*) and *MALATE SYNTHASE* (*OsMS*); and *PYRUVATE ORTHOPHOSPHATE DIKINASE B* (*OsPPDKB*) and *ASPARAGINE SYNTHASE 1* (*OsAS1*), whose encoded proteins are involved in amino acid remobilization (J. Liu *et al*., 2016). Conversely, deficiency in OsBBX11 resulted in salt hypersensitivity due to excessive accumulation of Na^+^ and K^+^ in shoots, revealing this protein as a positive regulator of ion homeostasis (Lei *et al*., 2023). In a similar way, the soybean *GmCOL1a* enhances salt and drought tolerance by modulating ion transport between roots and shoots. The underlying mechanisms involve the direct induction of the stress‐responsive genes *LATE EMBRYOGENESIS ABUNDANT PROTEIN* (*GmLEA*) and *PYRROLINE‐5‐CARBOXYLATE SYNTHASE* (*GmP5CS*), as well as the ABA‐mediated stomatal closure (C. Xu *et al*., 2023). Some other BBX proteins that modulate drought response also act by regulating ABA signaling. In apple (*Malus domestica*), MdBBX22 enhances drought tolerance by delaying ABA‐induced leaf senescence through the reduction in MdHY5‐mediated activation of *ABA INSENSITIVE 5* (*MdABI5*) transcription and the physical interaction with MdABI5, interfering with its ability to activate the Chl catabolic genes *MdSGR1* and *MdNYC1* (An *et al*., 2021b). Furthermore, under water‐limiting conditions, MdBBX7 directly activates drought‐responsive genes, including *ETHYLENE RESPONSE FACTOR 1* (*MdERF1*), *EARLY RESPONSIVE TO DEHYDRATION 15* (*MdERD15*) and *MdGLK1*; however, MdBBX7 is degraded by MYB30‐INTERACTING E3 LIGASE 1 (MdMIEL1) under water availability. Overexpression of *MdBBX7* increases the activity of the antioxidant enzymes peroxidase and catalase, elevates ABA levels and reduces H_2_O_2_ accumulation, conferring higher stress resilience (Chen *et al*., 2022).

In tomato, *SlBBX25* enhances salt tolerance by increasing antioxidant enzyme activities, improving water content and photosynthesis, thereby reducing malondialdehyde (MDA) and reactive oxygen species (ROS) levels. It directly induces stress‐protective genes, such as *PYRROLINE‐5‐CARBOXYLATE REDUCTASE* (*SlP5CR*) and *SMALL HEAT SHOCK PROTEINS* (*SlHSP21.6A* and *SlHSP26.2*; Ma *et al*., 2024). Allelic variation in *BBX18* among tomato species underscores its role in drought adaptation. The *SlBBX18* allele, from the drought‐sensitive cultivated tomato, encodes a truncated protein compared with drought‐tolerant wild species, *Solanum pennellii*. Although the full‐length SpBBX18 transcriptionally represses *ASCORBATE PEROXIDASE 1* (*SlAPX1*), the reduction in SlAPX1 activity caused by its dimerization with the truncated SlBBX18 is even stronger than the transcriptional repression, ultimately weakening the drought tolerance mechanisms of cultivated tomato (Li *et al*., 2024).

The conserved role of BBX proteins in drought response is also evident in other crops. The silencing of peanut (*Arachis hypogaea*) *AhBBX6* leads to reduced antioxidant enzyme activity and elevated MDA levels (Tang *et al*., 2024). Similarly, loss of StBBX24 function in potato diminished salinity tolerance, as reflected by reduced Na^+^/H^+^ antiporter transcript levels and lowered antioxidant enzyme activity following salt treatment (Kiełbowicz‐Matuk *et al*., 2022). In canola, BnBBX22.A07 enhances salinity tolerance by heterodimerizing with BnHY5.C09 to boost its activation of the *BnWRKY33.C03* promoter (Zhang *et al*., 2024). Conversely, in Chinese mustard, BjuBBX6 negatively regulates drought tolerance by lowering Chl levels and antioxidant activity through direct repression of *BjuGLK* and activation of *BjuABI2* (Feng *et al*., 2025). Finally, under normal conditions, CmBBX19 from *C. morifolium* dimerizes with CmABF3 suppressing its transcriptional activation of ABA‐responsive genes, such as *RESPONSIVE TO ABA 18* (*CmRAB18*). Under drought stress, ABA accumulation downregulates *CmBBX19*, relieving CmABF3 repression and inducing ABA‐responsive genes (Xu *et al*., 2020).

Several BBX genes have been characterized as regulators of extreme temperature tolerance, acting via distinct mechanisms. For example, the expression of *C‐REPEAT BINDING FACTORs* (*SlCBFs*), whose proteins bind cold‐ and dehydration‐responsive elements in target promoters to activate cold‐responsive genes, is upregulated by SlBBX17 and SlBBX31 (Song *et al*., 2023; Zhu *et al*., 2023). Interestingly, SlHY5 is stabilized by SlBBX17 and, together, they induce *SlCBFs*, while SlHY5 also upregulates *SlBBX31*. This induction is strengthened in domesticated tomato genotypes as consequence of a 27 bp deletion in the *SlBBX31* promoter. Under low‐temperature conditions, the upregulation of SlBBX25 induces the expression of genes associated with cold response, antioxidant activity, anthocyanin biosynthesis and starch catabolism. These molecular changes mitigate ROS and MDA accumulation, enhancing photosynthetic efficiency under stress (Ma *et al*., 2025). The overexpression of the potato *StBBX14* reduces leaf damage and electrolyte leakage under low temperature, although the underlying molecular mechanism remains uncharacterized (Zhang *et al*., 2025a).

The contrasting effects of tobacco (*Nicotiana tabacum*) NtBBX9 and NtBBX11 in response to low temperatures highlight the complex regulatory networks in which BBX factors participate to modulate stressful conditions. Both genes are upregulated by cold treatment; however, while NtBBX11 induces the expression of the cold‐responsive genes *NtCBFs*, *NtLEA14* and *LOW‐TEMPERATURE‐INDUCED 65* (NtLTI65), NtBBX9 represses them (S. Liu *et al*., 2023). Finally, under normal conditions, the activity of apple MdBBX37 is suppressed by MdMIEL1‐mediated ubiquitination and by the interaction with the jasmonic acid (JA) response repressors JASMONATE‐ZIM‐DOMAIN PROTEIN 1 (MdJAZ1) and MdJAZ2. Cold‐induced JA accumulation triggers the degradation of MdMIEL1 and JAZ proteins, releasing MdBBX37 to activate *MdCBF1* and *MdCBF4* expression, promoting cold tolerance (An *et al*., 2021a).

Although heat stress is increasingly relevant to crop improvement, studies on BBX regulation of high‐temperature responses have lagged behind. In tomato, the overexpression of *SlBBX17* enhances heat tolerance by stabilizing membranes, reducing ROS and upregulating the expression of *HEAT SHOCK FACTORS* (SlHSFs), *SlHSPs* and ROS detoxification genes (Xu *et al*., 2022a).

Beyond drought and extreme temperatures, BBX proteins are also involved in responses to light‐stress conditions, particularly SAS, often in a PIFs and/or HY5‐mediated manner. In *B. juncea*, BjuCOL13 is a negative regulator of shade avoidance that interacts with PHYTOCHROME A SIGNAL TRANSDUCTION 1 (BjuPAT1) factor under low R : FR light to suppress shoot branching (Muntha *et al*., 2019). In maize, DOUBLE B‐BOX ZINC FINGER PROTEIN 2 (ZmDBB2) acts as a central regulator of SAS. Under reduced R : FR light conditions, ZmPIF4 upregulates *ZmDBB2* expression, and ZmDBB2‐ZmPIF4 dimer synergistically promotes the expression of cell elongation‐related genes, such as *EXPANSIN 1* (*ZmEXPA1*). Under high R : FR light conditions, ZmDBB2 interacts with ZmHY5 to repress its ability to induce the GAs catabolic gene *ZmGA2OX4*, thus positively regulating growth (X. Wang *et al*., 2025). By contrast, tomato SlBBX20 negatively regulates SAS by inhibiting SlPIF4 activity through dimerization, thereby dampening the expression of *SlIAA29*, whose encoded protein downregulates the auxin‐repressor *SlIAA17* (Shiose *et al*., 2024).

Altogether, the accumulated evidence reveals the multifunctional role of BBX proteins in coordinating abiotic stress responses. By integrating hormonal signaling, antioxidant defense and gene expression regulation, BBXs emerge as promising targets for the genetic improvement of crops facing challenging environmental conditions.

### 2. Biotic stress response

Beyond abiotic stresses, plants constantly face pathogen infection and insect herbivory, which can strongly limit growth. These challenges activate phytohormone‐mediated defense pathways: salicylic acid (SA) primarily mediates immunity against biotrophic pathogens and sap‐sucking insects, whereas JA confers resistance to necrotrophic pathogens and chewing insects. Multiple molecular mechanisms regulate the crosstalk between light‐driven growth and defense, explaining why rapid growth often compromises immunity. This trade‐off is particularly problematic under the high‐density planting typical of extensive agriculture (Ballaré & Pierik, 2017). As BBX proteins become increasingly recognized for their roles in light signaling, their involvement in biotic stress responses is also gaining attention. (Fig. 3; Table 1).

In sweet potato (*Ipomoea batatas*), IbBBX24 enhances resistance to *Fusarium oxysporum* by upregulating and downregulating the JA‐responsive TF *MYELOCYTOMATOSIS ONCOGENE 2* (*IbMYC2*) and the JA response repressor *IbJAZ10* by directly binding to genes' regulatory regions. Additionally, IbBBX24‐IbJAZ10 heterodimerization alleviates the IbJAZ10‐mediated repression of IbMYC2, triggering a JA‐mediated response (Zhang *et al*., 2020). By contrast, tomato SlBBX25 reduces SlMYC2 activity by competing for binding to the transcriptional regulator MEDIATOR COMPLEX SUBUNIT 25 (SlMED25), thereby attenuating JA signaling and reducing the resistance to the necrotrophic fungus *Botrytis cinerea* (Luo *et al*., 2023). Conversely, SlBBX20 confers fruit resistance to this fungus by promoting the accumulation of protective compounds, such as flavonoids and steroidal glycoalkaloids (Shiose *et al*., 2024).

The overexpression of the hybrid bamboo (*Bambusa pervariabilis* × *Dendrocalamopsis grandis*) BDBBX21 led to elevated antioxidant enzyme activity and improved disease resistance against *Arthrinium phaeospermum* fungus (Y. Liu *et al*., 2024). Similarly, the cucumber (*Cucumis sativus*), CsCOL9 increases tolerance to the whitefly *Bemisia tabaci* by enhancing antioxidant enzyme activity and hydrogen peroxide levels (Xie *et al*., 2025).

BBXs also mediate defense through pathways other than JA signaling. For instance, in lily (*Lilium pumilum*), LpBBX28‐LpNAC48 dimer upregulates the expression of trichome formation‐associated TF *GLABRA3 LIKE* (*LpGL3L*) promoting trichome initiation and aphid resistance (Xin *et al*., 2025). Another case was described in chrysanthemum, where susceptibility to *Alternaria* sp. infection increased upon the silencing of *CmBBX32*, due to an attenuated SA response (B. Wang *et al*., 2025). Two BBX‐mediated resistance mechanisms against *Phytophthora* have been characterized. In potato, StBBX27 induces the expression of the defense‐related genes *StWRKY8* and *SOMATIC EMBRYOGENESIS RECEPTOR KINASE 2* (*StSERK2*) in response to *P. infestans* infection, thereby enhancing plant immunity. As a counter‐defense strategy, the oomycete effector Pi18609 interacts with StBBX27, increasing the affinity for the CONSTITUTIVE PHOTOMORPHOGENIC 1 (StCOP1) ubiquitin ligase, promoting StBBX27 ubiquitination and, subsequent, degradation (Sun *et al*., 2025). Finally, resistance against *P. capsici* in pepper (*Capsicum annuum*) is promoted by CaBBX14 through the upregulation of the SA response (Zhou *et al*., 2023). *CaBBX14* silencing reduces the expression of the SA biosynthetic gene *PHENYLALANINE AMMONIA LYASE 1* (*CaPAL1*) and, consequently, of the SA‐induced defense genes, such as *PATHOGENESIS RELATED 1* (*CaPR1*), *CaPR5* and *SYSTEMIC ACQUIRED RESISTANCE 8.2* (*CaSAR8.2*).

## VI. Specialized metabolism

Given the stress‐mitigating roles of specialized metabolites, such as flavonoids, anthocyanins and carotenoids in plants (Nawkar *et al*., 2024), along with their well‐documented health benefits in humans (Simsek & Whitney, 2024), enhancing the accumulation of these compounds in fruits and vegetables has become a major research priority.

**Fig. 4:**
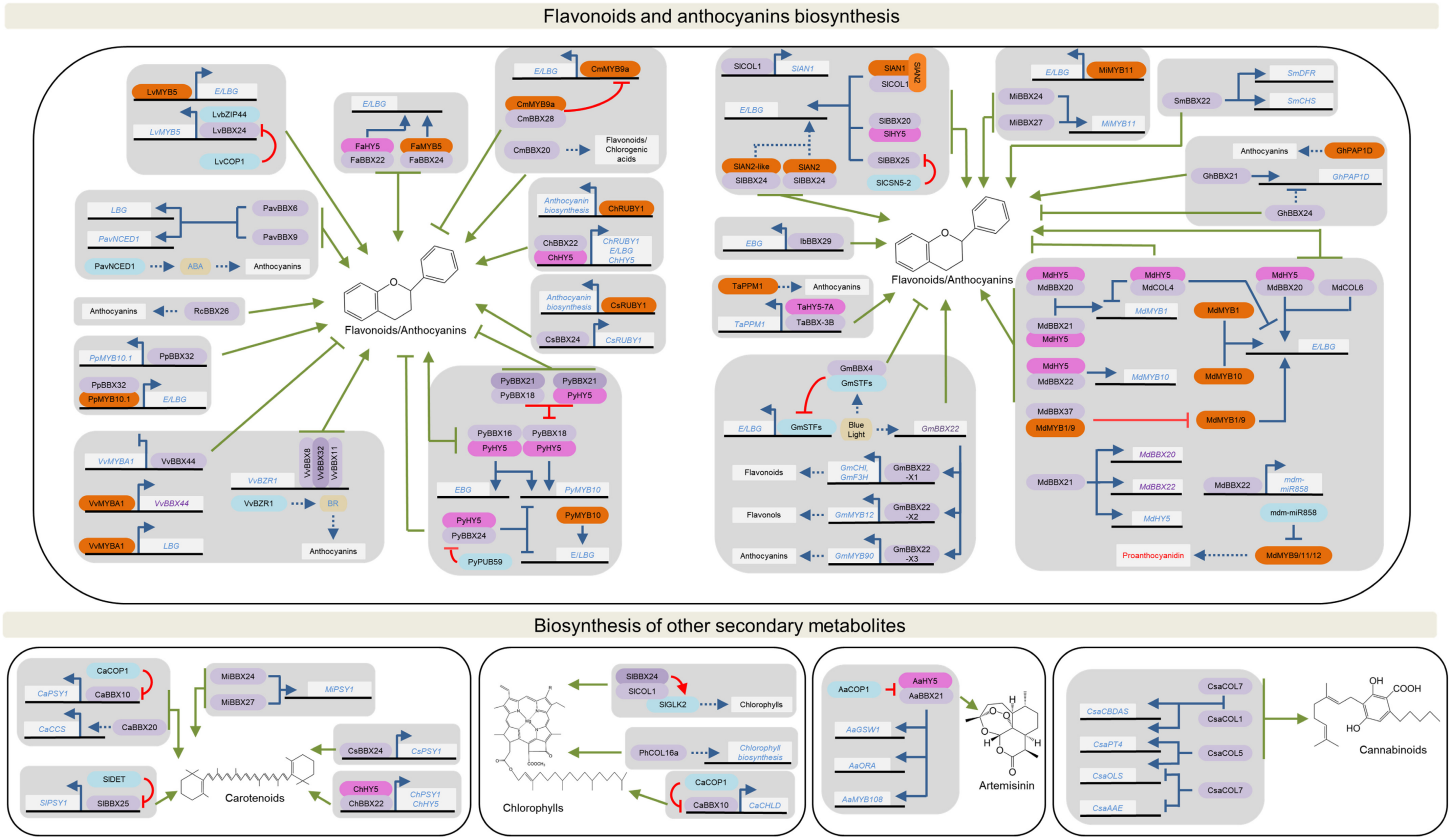
B‐box (BBX) genes that regulate specialized metabolism in crop species. The species are indicated by the genus and species initials. *Artemisia annua* (*Aa*), *Cannabis sativa* (*Csa*), *Capsicum annuum* (*Ca*), *Chrysanthemum morifolium* (*Cm*), *Citrus hindsii* (*Ch*), Citrus sinensis (*Cs*), *Fragaria × ananassa* (*Fa*), *Glycine max* (*Gm*), *Lilium* spp (*Lv*), *Malus domestica* (*Md*), *Mangifera indica* (*Mi*), *Petunia hybrida* (*Ph*), *Prunus avium* (*Pav*), *Prunus persica* (*Pp*), *Pyrus pyrifolia × Pyrus communis* (*Py*), *Rosa chinensis* (*Rc*), *Solanum lycopersicum* (*Sl*), *Solanum melongena* (*Sm*) and *Vitis vinifera* (*Vv*). Black lines and white boxes represent DNA and genes, respectively. E/LBG means early and late biosynthetic genes. BBX proteins are represented in purple, HY5 in pink, MYELOBLASTOSIS (MYBs) in orange, other proteins in blue and hormones in yellow boxes. Blue lines indicate transcriptional regulation, red lines indicate post‐translational regulation, and green lines indicate the effect of the BBX protein on the phenotype. Arrows represent positive regulation, and blunt‐ended arrows represent inhibition. Solid and dotted lines indicate direct or indirect effects, respectively. Both gene and hormone names are written in red or blue depending on whether they have a negative or a positive effect on the phenotype, respectively. Gene, protein and hormone abbreviations are detailed in the text.

Because light perception and signaling play pivotal roles in regulating specialized metabolism, and BBX proteins are key regulators of light responses, these proteins are increasingly recognized as major modulators of specialized metabolite synthesis and accumulation. They act either by directly activating biosynthetic genes or by regulating, transcriptionally and/or post‐translationally, TFs such as HY5 and the MYELOBLASTOSIS (MYBs), well‐known inducers of the flavonoid and anthocyanin biosynthesis (Fig. 4; Table 1). Such is the case of AtBBX20 that promotes anthocyanin synthesis by inducing *AtHY5* expression and by cooperatively upregulating the anthocyanin biosynthesis‐related target genes through dimerization with AtHY5 (Wei *et al*., 2016).

In tomato, while SlBBX20 alone induces the expression of *CHALCONE SYNTHASE 1* (*SlCHS1*), *CHALCONE ISOMERASE* (*SlCHI*), *FLAVANONE 3‐HYDROXYLASE* (*SlF3H*) and *FLAVONOL SYNTHASE* (*SlFLS*), its heterodimerization with SlHY5 intensifies flavonoid biosynthetic genes transcription during fruit ripening (Shiose *et al*., 2024). In vegetative organs, the direct binding of SlBBX25 to the promoter of the biosynthetic gene *DIHYDROFLAVONOL 4‐REDUCTASE* (*SlDFR*) induces the accumulation of anthocyanins in tomato plants. In this system, SlBBX25 protein stability is modulated by ubiquitination via COP9 SIGNALOSOME SUBUNIT 5–2 (SlCSN5‐2; Luo *et al*., 2021). SlCOL1 (SlBBX3) promotes anthocyanin accumulation to counteract ROS accumulation under short‐day and low‐temperature conditions in tomato leaves. It induces the *ANTHOCYANIN 1* (*SlAN1*) MYB TF expression, and subsequently, the formation of the SlCOL1‐SlAN1‐SlAN2 complex upregulates anthocyanin biosynthetic genes (Liu *et al*., 2025). In both tomato seedlings and fruits, SlBBX24 promotes anthocyanin accumulation by interacting with the ANTHOCYANIN 2 (SlAN2) and SlAN2‐LIKE TFs (He *et al*., 2023).

In apple fruits, MdMYB1 and MYB10 are the main activators of anthocyanin biosynthetic genes. *MdMYB1* is activated by MdBBX20‐MdHY5 (Fang *et al*., 2019b) and MdBBX21‐MdHY5 (Zhang *et al*., 2021) dimers, while *MdMYB10* expression is upregulated by MdBBX22‐MdHY5 (An *et al*., 2019). Moreover, MdBBX21 directly activates the expression of *MdBBX20*, *MdBBX22* and *MdHY5* (Zhang *et al*., 2021). Additionally, MdBBX22 upregulates the miRNA mdm‐miR858 that post‐transcriptionally represses MdMYB9/11/12, which are TFs that enhance proanthocyanidin biosynthesis. Since anthocyanin and proanthocyanidin compete for the same precursors, MdBBX22 ultimately enhances anthocyanin accumulation (Zhang *et al*., 2022). Conversely, MdBBX37 diminishes anthocyanin synthesis by inhibiting the transcriptional activity of MdMYB1/9 over *MdDRF*, *ANTHOCYANIDIN SYNTHASE* (*MdANS*) and *UDP‐GALACTOSE FLAVONOID 3‐O‐GALACTOSYLTRANSFERASE* (*MdUFGT*) via heterodimerization (An *et al*., 2020). MdCOL4 together with MdHY5 also inhibit the expression of MdMYB1 and the late anthocyanin biosynthetic genes *MdANS* and *MdUFGT* (Fang *et al*., 2019a). Finally, MdCOL6 also induces the transcription of *MdANS* and *MdUFGT* in fruits (Wang *et al*., 2023b). Together, these data demonstrate a highly intricate BBX‐mediated network regulating anthocyanin accumulation in apple fruits.

The regulation of HY5 activity by BBX proteins has also been reported in other species. In strawberry (*Fragaria* × *ananassa*), FaBBX22 interacts with FaHY5 to synergistically promote the expression of anthocyanin biosynthetic genes (Liu *et al*., 2022). Likewise, anthocyanin accumulation is enhanced by the FaMYB5–FaBBX24 dimer, which strengthens FaMYB5 activation of its target genes in the fruits (Zhang *et al*., 2023). In citrus (*Citrus hindsii*), the ChBBX22‐ChHY5 dimer promotes fruit anthocyanin accumulation not only by activating the promoters of the biosynthetic genes *FLAVONOID 3′‐HYDROXYLASE* (*ChF3′H*), *ChDFR* and *ChANS* but also by inducing the expression of the MYB TF *ChRUBY1* responsible for the induction of the anthocyanin biosynthetic genes (Fu *et al*., 2024). By a similar mechanism, during blood orange (*Citrus sinensis*) ripening, CsBBX24 also promotes the expression of *CsRUBY1* (Fu *et al*., 2025).

In peach fruits, PpBBX32 promotes anthocyanin accumulation by directly upregulating the TF PpMYB10.1 and by enhancing its ability to activate anthocyanin biosynthetic genes through heterodimerization (Huang *et al*., 2024). Similarly, in mango (*Mangifera indica*) fruit peel, MiBBX24‐ and MiBBX27‐dependent activation of *MiMYB11* mediates the blue light‐induced anthocyanin accumulation (Pan *et al*., 2025). In eggplant (*Solanum melongena*), SmBBX22 directly induces *SmCHS* and *SmDRF* to promote anthocyanin accumulation (J. Li *et al*., 2025); whereas the palm leaf raspberry (*Rubus chingii*) RcBBX26 was also reported as an inductor of this pathway during fruit ripening (Xu *et al*., 2024).

In grape (*Vitis vinifera*), VvBBX44 is a negative regulator of anthocyanin accumulation that represses the expression of *VvMYBA1*, which in turn induces *VvUFGT* expression. Although VvBBX44 and VvMYBA1 do not interact, they form a feedback inhibitory loop to prevent pigments overaccumulation in grape berries (W. Liu *et al*., 2023). By contrast, during veraison, VvBBX8, VvBBX11 and VvBBX32 physically interact and induce the expression of their direct target *BRASSINAZOLE‐RESISTANT 1* (*VvBZR1*), enhancing the BR‐triggered accumulation of anthocyanins in the berries (Qiu *et al*., 2025). Another case of BBX‐mediated anthocyanin accumulation via hormonal pathways has been reported in sweet cherry (*Prunus avium*). PavBBX6 and PavBBX9 induce light and ABA‐mediated anthocyanin accumulation by directly binding to the promoters of *PavUFGT* and *9‐CIS‐EPOXYCAROTENOID DIOXYGENASE 1* (*PavNCED1*), which encode key enzymes in anthocyanin and ABA biosynthesis, respectively (Wang *et al*., 2023a).

In pear fruits, both PyBBX16‐PyHY5 and PyBBX18‐PyHY5 heterodimers upregulate *PyMYB10*, inducing anthocyanin accumulation (Bai *et al*., 2019a,b). Adding an extra layer of control, PyBBX21 interacts with both PyHY5 and PyBBX18, limiting the formation of the PyHY5‐PyBBX18 transcription activator complex (Bai *et al*., 2019b). Additionally, PyBBX24‐PyHY5 dimer represses *PyUFGT, PyCHS* and *PyMYB10* expression. This negative regulatory effect is alleviated through the PLANT U‐BOX TYPE E3 UBIQUITIN LIGASE 59 (PyPUB59)‐mediated PyBBX24 degradation (Yang *et al*., 2024; Li *et al*., 2025a). Different *PyBBX24* alleles with nonsense mutations were identified in several varieties. In contrast to the wild‐type PyBBX24, the truncated proteins promote anthocyanin biosynthesis (Yang *et al*., 2024). Similarly, in cotton, a mutant allele of *GhBBX24* (*GhDR*) results in a shortened protein that fails to repress the expression of *PRODUCTION OF ANTHOCYNIN PIGMENT 1D* (*GhPAP1D*) MYB TF, upregulating anthocyanin biosynthetic genes. Additionally, GhBBX21 is a positive regulator of *GhPAP1D* expression, inducing the pigment accumulation (X. Wang *et al*., 2023; Li *et al*., 2025b).

An interesting splicing‐dependent regulatory module involving GmBBX22 and different MYB TFs is required for the blue light‐induced biosynthesis of anthocyanins and flavonols in soybean seedlings (Zhan *et al*., 2025). Under blue light, *GmBBX22* transcripts are alternatively spliced, producing 3 isoforms: X1, X2 and X3. The full‐length X1 directly induces *GmCHI* and *GmF3H* transcription, while the truncated isoforms X2 and X3 induce the expression of *GmMYB12* and *GmMYB90*, respectively. This specific regulation differentially impacts the metabolic flux partitioning between flavonols and anthocyanins as GmMYB12 and GmMYB90 activate *GmFLS* and *GmDFR*, respectively (Zhan *et al*., 2025). Moreover, in seeds, GmBBX4 dimerizes with blue light‐induced TFs SOYBEAN TGACG‐MOTIF BINDING FACTOR 1 (STF1) and STF2, impairing their transcriptional activation of flavonoid biosynthetic genes (Song *et al*., 2024c).

Relatively fewer studies have investigated the roles of BBXs in controlling specialized metabolism in other organs than fruits and leaves. In flowers of *C. morifolium*, CmBBX20 was implicated as a positive mediator of light‐triggered accumulation of flavonoids and chlorogenic acid (CGA; Lu *et al*., 2024). Conversely, CmBBX28‐CmMYB9a dimer reduces the ability of CmMYB9a to activate *CmCHS*, *CmDFR* and *CmUFGT* (L. J. Zhou *et al*., 2025). A more complex regulatory module was identified during anthocyanin accumulation in *Lilium* spp. flowers. Light‐induced LvBBX24 interacts with the bZIP TF LvbZIP44 to promote *LvMYB5* expression, which subsequently activates anthocyanin biosynthetic genes. In darkness, this pathway is suppressed through LvCOP1‐mediated degradation of LvBBX24 (Gao *et al*., 2024). In wheat (*Triticum aestivum*) grains, TaBBX‐3B interacts with TaHY5‐7A to activate *PURPLE PERICARP‐MYB 1* (*TaPPM1*) TF, leading to the accumulation of anthocyanins in the pericarp (Jiang *et al*., 2023). Finally, in sweet potato, overexpression of *IbBBX29* increases flavonoid accumulation in both leaves and storage roots by directly activating the flavonoid biosynthetic genes *IbCHS*, *IbCHI1* and *IbF3203'H* (Gao *et al*., 2023).

Besides promoting anthocyanin accumulation in the mango fruit peels, MiBBX24 and MiBBX27 mediate blue light‐induced carotenoid accumulation in the fruit flesh by directly activating the expression of the biosynthetic gene *PHYTOENE SYNTHASE* (*MiPSY*; Pan *et al*., 2025). Similarly, SlBBX25 and CaBBX10, both subjected to DE‐ETIOLATED 1‐ (SlDET1) and CaCOP1‐mediated ubiquitination, directly upregulate *PSY*, leading to carotenoid accumulation in tomato and pepper fruits, respectively (Xiong *et al*., 2019; J. Wang *et al*., 2025). In addition, *CaBBX20* silencing reduced carotenoid content in pepper fruits due to the downregulation of the *CAPSANTHIN‐CAPSORUBIN SYNTHASE* (*CaCCS*; Ma *et al*., 2023). Interestingly, during citrus fruit ripening, ChBBX22 and CsBBX24 also activate the *PSY1* promoter, and in the case of ChBBX22, this activation is further enhanced through dimerization with ChHY5 (Fu *et al*., 2024, 2025).

The influence of BBXs regulation on Chl biosynthesis has been explored in petunia (*Petunia hybrida*) flowers, where *PhCOL16a* overexpression activates Chl biosynthetic genes (Ohmiya *et al*., 2019). In addition, the interaction of SlCOL1 and SlBBX24 with SlGLK2 stabilizes this master regulator of chloroplast development, leading to enhanced Chl accumulation in immature tomato fruits. In the absence of SlCOL1, SlBBX24 is unable to interact with SlGLK2, facilitating ubiquitin‐mediated degradation of SlGLK2, limiting Chl biosynthesis in the fruits (Cui *et al*., 2025). CaBBX10 also promotes Chl biosynthesis in green pepper fruits by activating the transcription of the *MAGNESIUM CHELATASE SUBUNIT D* (*CaCHLD*; J. Wang *et al*., 2025).

The *Artemisia annua* AaBBX21 was the first BBX identified as a regulator of terpenoid biosynthesis. The accumulation of the sesquiterpene lactone artemisinin is enhanced by the activity of the AaBBX21‐AaHY5 dimer that synergistically upregulates the transcription of *GLANDULAR TRICHOME‐SPECIFIC WRKY 1* (*AaGSW1*), *OVEREXPRESSION OF REGULATORY ACTIVITY* (*AaORA*) and *AaMYB108* genes. The interaction between AaBBX21 and AaHY5 with the ubiquitin ligase AaCOP1 reduces the abundance of both TFs, explaining the reduction of artemisinin content in the dark (He *et al*., 2024).

Finally, cannabis (*Cannabis sativa*) CsaCOL1, CsaCOL5 and CsaCOL7 were identified as regulators of key cannabinoid biosynthetic genes. While CsaCOL1 and CsaCOL7 induce the expression of *GERANYLDIPHOSPHATE:OLIVETOLATE GERANYLTRANSFERASE 4* (*CsaPT4*) and *CANNABIDIOLIC ACID SYNTHASE* (*CsaCBDAS*), CsaCOL5 upregulates *OLIVETOL SYNTHASE* (*CsaOLS*) and *CsaPT4*. Strikingly, CsaCOL7 represses *ACYL‐ACTIVATING ENZYME* (*CsaAAE*) and *CsaOLS*, indicating a dual role fine‐tuning cannabinoid biosynthesis (M. Gao *et al*., 2025).

## VII. Conclusions and perspectives

The knowledge about the function of BBX proteins has grown substantially in recent years. The state‐of‐the‐art on *A. thaliana* and crop BBXs reveals an evolutionarily conserved mode of action across species. As covered at length in the text, in addition to regulating gene expression by directly binding to the promoters of their target genes, BBXs modulate the activity of other TFs, including the core light‐signaling regulators HY5 and PIFs. Furthermore, ubiquitin ligase complex–mediated post‐translational degradation is another regulatory level of BBX proteins.

Although significant advances have been made, numerous aspects still require refinement to achieve a comprehensive understanding of the multilayer functions of the BBX proteins and to translate this knowledge into effective strategies for crop improvement.

The phenotypic information obtained from *BBX* overexpression lines must be interpreted with caution. For instance, in tomato, overexpression of *SlBBX17* impairs plant growth (Xu *et al*., 2022a,b), SlBBX24 affects fruit size (Cui *et al*., 2022), whereas SlBBX25 alters fruit number (Luo *et al*., 2023), and the accumulation of carotenoids (Xiong *et al*., 2019) and anthocyanins (Luo *et al*., 2023). Notably, these phenotypes were detected only in overexpressing lines and were absent in the corresponding loss‐of‐function mutants. Such discrepancies may arise from ectopic or supraphysiological gene expression driven by constitutive promoters and/or functional redundancy among BBX paralogs.

**Fig. 5:**
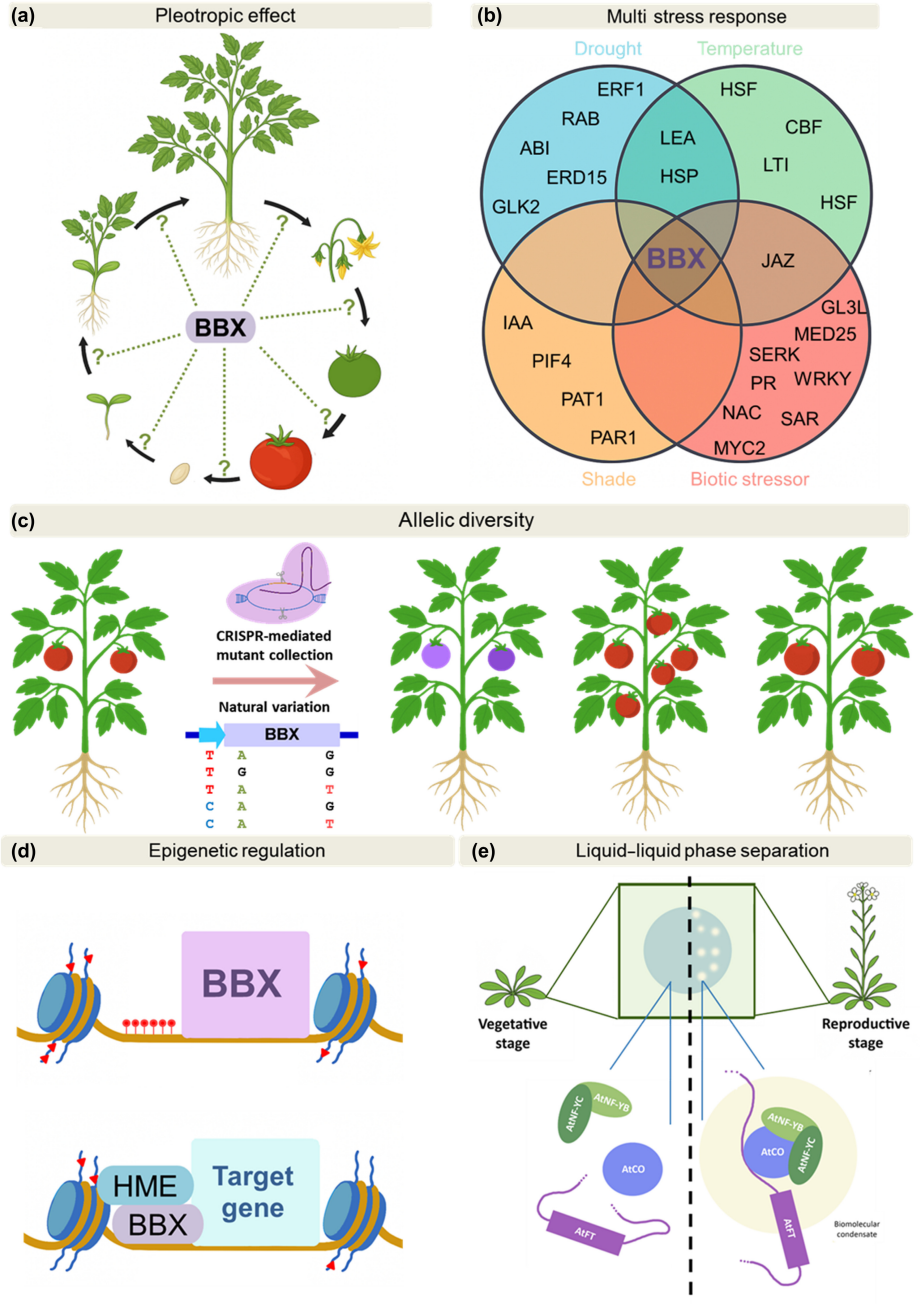
Future perspectives. A roadmap for elucidating the B‐box (*BBX*) genetic diversity, regulatory mechanisms and multilayered functions to facilitate future BBX‐based breeding strategies. (a) Comprehensive analysis of *BBX* effects throughout the plant life cycle is essential to enable precise time‐ and organ‐specific manipulations and to prevent undesirable outcomes. (b) Current evidence indicates that individual BBX proteins mediate several stress responses and coordinate defense mechanisms with developmental programs by modulating the activity of regulatory factors. However, there remains a knowledge gap regarding the role of BBXs in the molecular signaling crosstalk triggered by multi‐stress stimuli. Protein abbreviations are detailed in the text. (c) The generation and functional characterization of mutant collections targeting single and multiple BBX genes (e.g. Clustered Regularly Interspaced Short Palindromic Repeats‐mediated mutants) together with the exploration of their natural allelic diversity will reveal underexplored genetic resources for the modulation of important agronomical traits in crops. (d) Although evidence suggests that BBX genes are regulated by and also regulate target genes through epigenetic mechanisms, a substantial knowledge gap remains in this area. HME indicates histone‐modifying enzymes. (e) Recently, it has been reported that the AtCO‐mediated induction of *AtFT*, which triggers the vegetative‐to‐reproductive program transition in *A. thaliana*, is not only driven by its interaction with AtNF‐YB and AtNF‐YC, but also by the proper stoichiometry of these three proteins, determining the formation of a functional biomolecular condensate within the cell nucleus. Further research on how BBX‐containing protein complexes are regulated through liquid–liquid phase separation will enhance our understanding of the regulatory landscape modulated by BBX proteins.

The few reports that have investigated the function of individual BBXs across the entire plant life cycle have consistently revealed their pleiotropic effects. For instance, tomato *Slbbx26* displays accelerated flowering and delayed fruit ripening (Moreira *et al*., 2025). Similarly, *Citrus hindsii* ChBBX22 inhibits internode elongation, while promoting anthocyanin and carotenoid accumulation in fruits (Fu *et al*., 2024). However, most reports characterize BBX function in the context of isolated traits or developmental stages. This fragmented information not only limits our understanding of BBX function, but also overshadows the potential existence of functional orthology within phylogenetic orthologous genes. Besides, the establishment of evolutionary relationships among BBXs from different species is hindered by phylogenetic analyses that rely on alignment and tree reconstruction algorithms lacking methodological rigor and branch support. The analysis of the available information for *A. thaliana* and tomato shows that, while the studies for AtBBXs are almost restricted to flowering time and light responses, SlBBXs were investigated in the context of other agronomically appealing processes, such as the regulation of fruit yield, stress responses and specialized metabolism (Fig. 1; Table 1). Based on current knowledge, only two cases of phylogenetic and functional orthology have been established between AtBBXs and SlBBXs: SlBBX25‐AtBBX20 (both positive regulators of specialized metabolism), and SlBBX1‐AtBBX1 (both flowering time regulators, albeit in opposite directions; Fig. 1; Table 1). In light of this scenario, comprehensively analyzing the roles of individual BBXs throughout the plant life cycle is paramount for assessing the impacts of their genetic manipulation for crop improvement. Based on this knowledge, the potential of BBX‐based breeding strategies can be further exploited by designing temporal‐ and organ‐specific manipulations to avoid undesirable trade‐offs (Fig. 5a).

Moreover, BBXs are increasingly recognized as key regulators of responses to diverse environmental stresses. Notably, there are examples of individual BBX regulating more than one stress‐response pathway. For example, in tomato, SlBBX20 is required not only for the shade avoidance response but also for the resistance to *Botrytis cinerea* (Shiose *et al*., 2024). The current evidence derived from single‐stress experiments does not reflect field conditions in which several stresses occur simultaneously. Thus, further studies using multi‐stress experimental approaches are needed to elucidate BBX role within the molecular signaling crosstalk (Fig. 5b).

As mentioned above, recent studies indicate that natural allelic variation in *BBX* genes regulates key agronomic traits, representing an underexplored resource for biotechnological applications (Fig. 5c). However, the pleiotropic nature of BBX proteins, and the fact that several family members interact with the same regulatory partners (*e.g*. HY5 and PIFs), suggests that functional redundancy may occur within the family. While the roles of individual *BBX* genes were characterized through single‐gene interventions, relatively few studies have examined redundancy among paralogs. Notably, to date, all reported cases of redundancy correspond to ‘partial redundancy’, as the phenotypes of single mutants are enhanced when combined in double mutants, indicating additive effects. Examples include AtBBX24/25 in shade‐induced hypocotyl elongation (Gangappa *et al*., 2013), and AtBBX20/21/22 (Bursch *et al*., 2020) and AtBBX22/23 (X. Zhang *et al*., 2017) during seedling photomorphogenesis. In rice, OsCOL4/13 also exhibit partial redundancy flowering time repressors (Sheng *et al*., 2016).

Thus, developing and systematically characterizing single and multiple BBX mutant collections will elucidate the regulatory complexity of this protein family and be crucial for translating mechanistic insights into biotechnological strategies (Fig. 5c).

Epigenetic regulation is another aspect of BBX biology that has lagged behind. In pear, analysis of DNA methylation during light‐induced anthocyanin accumulation led to the identification of a *BBX* gene whose promoter undergoes light‐dependent demethylation (Liu *et al*., 2022). In *A. thaliana*, the positive regulator of photomorphogenesis *AtBBX22* is transcriptionally repressed in darkness through HISTONE DEACETYLASE 19–mediated histone deacetylation (Jing *et al*., 2021). Moreover, transcription of the flowering inducer AtCO requires the removal of the repressive histone mark H3K9me2 by the demethylase JUMONJI 28 (AtJMJ28; Hung *et al*., 2021). Subsequently, AtCO interacts with the chromatin remodeling factor PICKLE (AtPKL), enhancing chromatin accessibility at the *AtFT* locus through increased histone H3 acetylation (Jing *et al*., 2019). AtCO also interacts with the histone‐methylation reader AtMRG2, which recognizes H3K4me and H3K36me, reinforcing AtCO binding to the *AtFT* promoter (Bu *et al*., 2014). Furthermore, AtCO promotes transcription of the floral integrator *SUPPRESSOR OF OVEREXPRESSION OF CONSTANS 1* (*AtSOC1*) by recruiting NUCLEAR FACTOR Y (NF‐Y) to its promoter, leading to histone demethylation and consequent transcriptional activation (Hou *et al*., 2014). Collectively, these findings demonstrate that epigenetic regulation operates both upstream and downstream of BBX proteins, influencing their expression and shaping the transcriptional output of their target genes. This underscores the need for deeper investigation into how BBXs and epigenetic mechanisms jointly orchestrate gene expression (Fig. 5d).

**Fig. 6:**
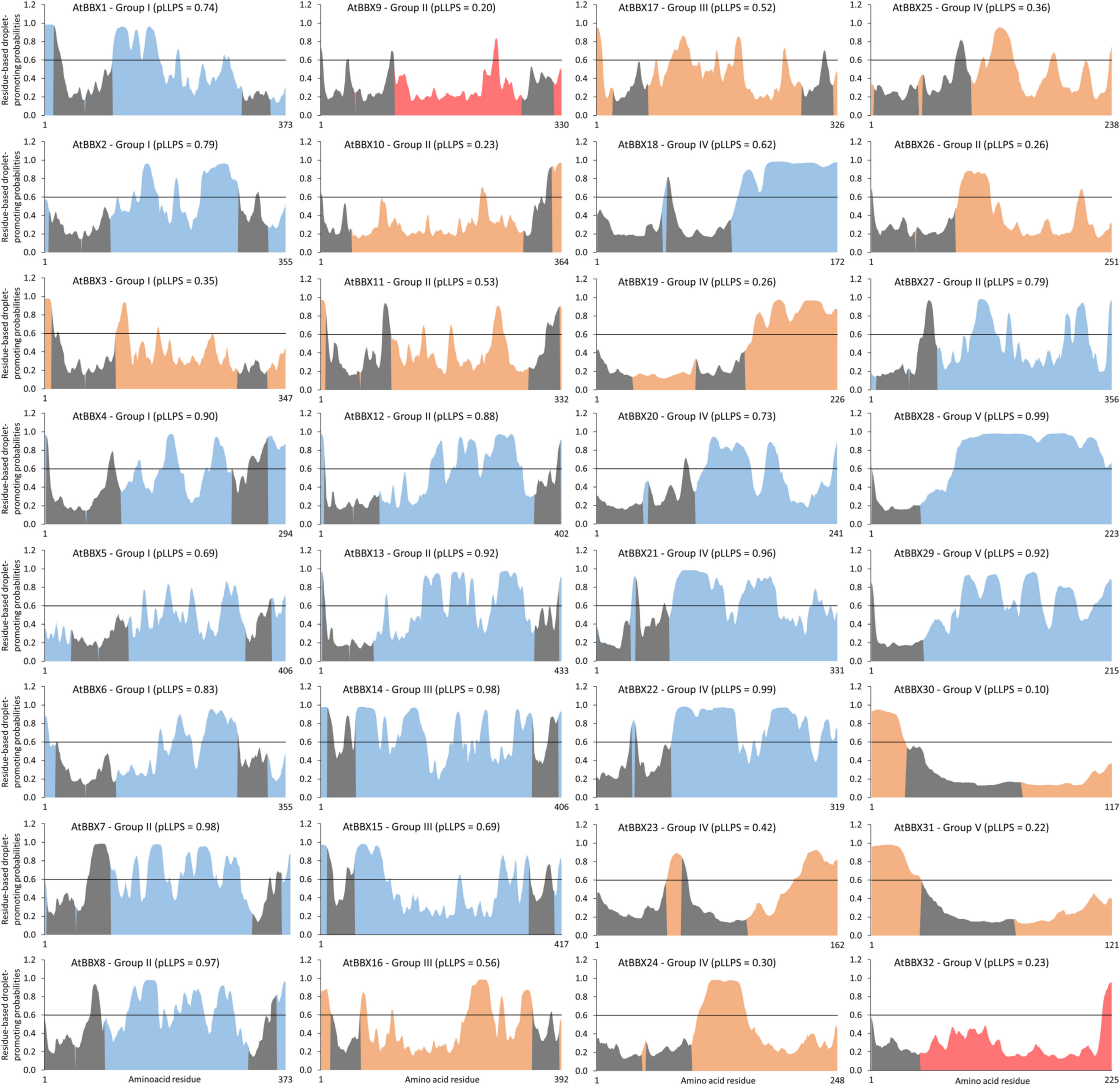
Probability of AtBBXs to undergo liquid–liquid phase separation. Graphical representation of the estimated probability of each amino acid residue to promote liquid–liquid phase separation (threshold = 0.6). The overall probability of spontaneous liquid–liquid phase separation (pLLPS) for each protein is indicated along with the corresponding protein name. Proteins whose chart is colored in blue are classified as droplet‐drivers (pLLPS ≥ 0.60), which can spontaneously undergo LLPS; those in orange are droplet‐clients (pLLPS < 0.6, but containing droplet‐promoting regions) that can compose biomolecular condensates through the interaction with droplet‐driver proteins, while in red are the proteins unlikely to undergo liquid–liquid phase separation. The gray areas indicate the domains (B‐box and CCT) within each protein. The prediction was performed with FuzDrop (Hatos *et al*., 2022). BBX, B‐box; CCT, CONSTANS, CONSTANS‐like and TIMING OF CAB1.

Finally, a few reports have described the same BBX differentially regulating the same biological process or metabolic pathway in different physiological contexts or organs. In barley, HvCO1 and HvCO2 regulate flowering by inhibiting or inducing HvFT1 before or after vernalization, respectively (Mulki & von Korff, 2016). The tomato SlBBX26 impairs SlPIF4 activity by heterodimerization, preventing GAs accumulation by downregulating GA biosynthetic genes in internodes and upregulating GA catabolic genes in fruits (Moreira *et al*., 2025). At first glance, these observations may appear contradictory; however, the repertoire of TFs in the nucleus varies across different cell types and developmental stages, leading to the formation of diverse biomolecular complexes. These protein–DNA complexes aggregate into membraneless compartments known as biomolecular condensates, which regulate gene expression by modulating transcriptional activity, chromatin remodeling, RNA processing, protein transport and degradation. Droplet condensates are formed through liquid–liquid phase separation (LLPS) driven by the liquid‐like properties of some of their protein constituents. Several studies have linked LLPS‐derived condensates to the regulation of plant developmental processes and stress responses (Kim *et al*., 2021; Q. Liu *et al*., 2024). In this sense, it was recently characterized that AtCO undergoes LLPS, creating condensates together with AtNF‐YB, AtNF‐YC and *AtFT* promoter region. The proper formation of this cluster is essential for the AtCO‐mediated activation of *AtFT* expression and, consequently, floral transition (Huang *et al*., 2025; Fig. 5e). This is the only experimental evidence of a BBX acting in LLPS opens up a promising field of study for further research. In fact, we predicted the overall probability to undergo LLPS, revealing that 18 AtBBXs are high‐confidence droplet drivers capable of spontaneously undergoing LLPS, whereas 12 AtBBXs are droplet clients that assemble into biomolecular condensates through interactions with droplet drivers (Fig. 6). Notably, this predicted condensate‐forming capacity does not correlate with either protein domain topology or DNA‐binding activity (Table S3). It is worth mentioning that the proper condensate functionality depends on the correct stoichiometry of its constituents; thus, their abundance is tightly regulated. This finding unveils an uncharted area for deciphering the molecular mechanisms governing the function of this multigene family.

**Fig. 7:**
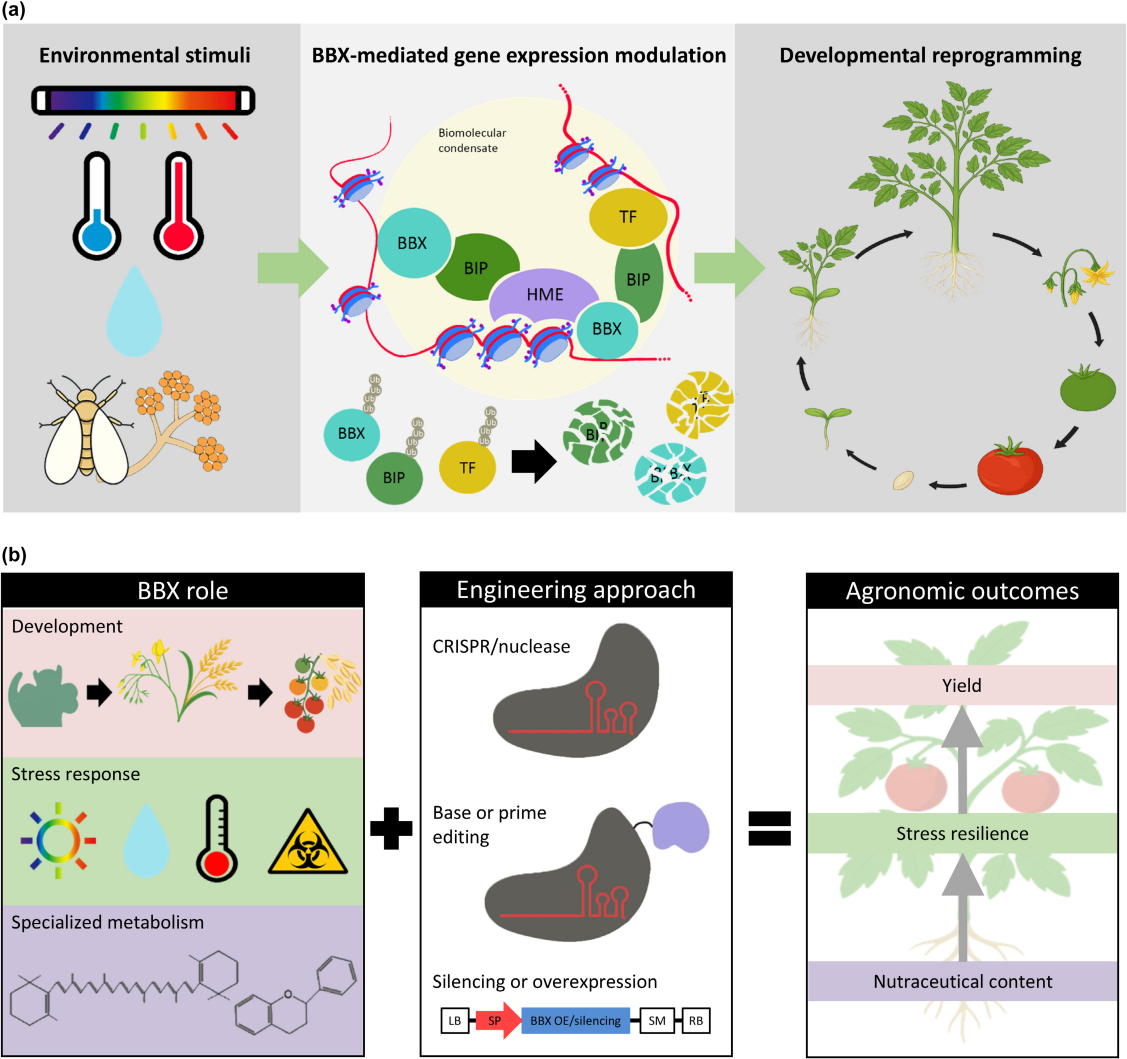
B‐box (BBX) proteins bridge environmental stimuli and developmental reprogramming, being candidates for crop breeding strategies. (a) Upon perception of environmental cues, including light intensity and quality, temperature, water availability and biotic stress, BBX proteins initiate adaptive reprogramming of gene expression. This regulation occurs through the direct binding of BBX proteins to promoter regions of target genes and/or through the assembly of multiprotein complexes comprising BBX‐interacting proteins (BIPs), histone‐modifying enzymes (HMEs) and additional transcription factors (TFs). HY5 and PIFs have been extensively reported as BIPs, being key hubs in BBX mode of action. These interactions can be facilitated by the formation of biomolecular condensates whose composition and dynamics are specific to tissue type, environmental context and developmental stage. The abundance of individual condensate components is tightly controlled at both transcriptional and post‐translational levels (*e.g.* via ubiquitin‐mediated degradation), as proper condensate function depends on precise stoichiometric balance. Collectively, this BBX‐centered signaling framework enables the plant developmental program to be dynamically reconfigured in accordance with prevailing environmental conditions. (b) As the functions of BBX proteins are more precisely defined, biotechnological interventions can be developed using genetic engineering approaches. Genome editing tools, such as CRISPR/nuclease systems, can be employed to generate null alleles of *BBX* genes acting as negative regulators of desirable traits. In addition, advanced CRISPR‐based strategies, including base editing and prime editing, allow precise modification of specific gene regions. These approaches enable the fine‐tuning of BBX gene regulation through targeted alterations in promoter sequences, the modification of BBX‐coding regions to disrupt interactions with repressor proteins, or the engineering of BBX downstream target genes to enhance or reduce BBX binding to their promoters and/or protein products. Furthermore, transgenic strategies using cell‐type‐ or stimulus‐specific promoters can achieve precise overexpression or silencing of *BBX* genes, allowing spatially and temporally controlled modulation of BBX activity. Collectively, these biotechnological strategies hold substantial potential for improving plant yield, development, stress resilience and nutraceutical content. LB, T‐DNA left border; RB, T‐DNA right border; SP, specific promoter; SM, selective marker.

In conclusion, BBX proteins are key factors that enable plants to integrate environmental signals into developmental programs (Fig. 7a). Continued research will uncover the full extent of BBX functional diversity and the variety of molecular mechanisms underlying the multiple regulatory levels at which they operate, providing a foundation for the development of precise biotechnological strategies. Then, to improve crop performance and stress resilience under changing environmental conditions, specific BBX genes can be targeted in particular organs and/or physiological processes. Their activity can be attenuated or enhanced using modern genetic modification tools, such as promoter or gene sequence editing, gene silencing or overexpression (Fig. 7b).

## Competing interests

None declared.

## Disclaimer

The New Phytologist Foundation remains neutral with regard to jurisdictional claims in maps and in any institutional affiliations.

## Supporting information


**Table S1** Functions of AtBBX loci.
**Table S2** Genome‐wide identification of BBX gene family.
**Table S3** AtBBXs DNA interaction.
Please note: Wiley is not responsible for the content or functionality of any Supporting Information supplied by the authors. Any queries (other than missing material) should be directed to the *New Phytologist* Central Office.
